# A questionnaire-based model to prioritize additional support during HIV testing encounters among men who have sex with men: temporal and geographic external validation

**DOI:** 10.3389/fpubh.2026.1944057

**Published:** 2026-10-16

**Authors:** Jian Zhao, Yazhou Yang, Ping Zhu, Shilin Zheng, Xiaowei Xu, Xiaoyi Zhou

**Affiliations:** 1 Department of Information Management, Nantong Center for Disease Control and Prevention, Nantong, Jiangsu, China; 2 Office of Comprehensive Affairs, Nantong Center for Disease Control and Prevention, Nantong, Jiangsu, China; 3 Department of HIV/AIDS and STI Prevention and Control, Nantong Center for Disease Control and Prevention, Nantong, Jiangsu, China; 4 Department of Medical and Preventive Care Integration, Nantong Center for Disease Control and Prevention, Nantong, Jiangsu, China

**Keywords:** external validation, HIV testing, men who have sex with men, prediction model, sentinel surveillance, service prioritization

## Abstract

**Background:**

HIV sentinel surveillance collects behavioral and service-use information from men who have sex with men (MSM), but these data are mainly used for routine reporting. We assessed whether pre-test questionnaire data could help prioritize testing encounters for optional additional support.

**Methods:**

We conducted a retrospective prediction modeling study using de-identified MSM surveillance records. Data from Nantong collected in 2020–2023 were used for model development, and records from 2024–2025 were used for temporal validation. Surveillance records from an external city in Shandong Province were used for geographic external validation. The final model was a calibrated stacked ensemble combining penalized logistic regression, random forest, and HistGradientBoosting. We evaluated the complete modeling and calibration workflow using nested cross-fitting. Model selection, calibration, and threshold selection used development data only.

**Results:**

The development cohort included 1,585 encounters and 73 confirmed HIV-positive events. Temporal validation included 803 encounters and 29 confirmed HIV-positive events, while geographic external validation included 1,622 encounters and 52 confirmed HIV-positive events. AUROC values were 0.963 (95% CI 0.930–0.990) and 0.974 (95% CI 0.955–0.989) in temporal and geographic validation, respectively. The corresponding AUPRC values were 0.696 (95% CI 0.536–0.880) and 0.746 (95% CI 0.652–0.842). At the locked threshold, sensitivity was 75.9 and 86.5%. Selecting the highest-scoring 10% of encounters captured 86.2 and 88.5% of confirmed events. Calibration slopes were 1.530 and 1.414, respectively. The model overestimated overall risk in geographic validation.

**Conclusion:**

Pre-test questionnaire data supported the ranking of HIV testing encounters in both temporal and geographic validation. The model may help services plan optional counseling, prevention assessment, peer support, or follow-up when service capacity is limited. The scores should not be treated as exact individual probabilities. They should not be used for HIV diagnosis or to restrict access to testing or other standard services. Prospective evaluation and monitoring are needed before routine use.

## 1 Introduction

Men who have sex with men (MSM) remain an important population in HIV prevention. Key populations and their partners account for a large share of new HIV infections, although the pattern varies across regions (1). HIV and syphilis also remain important health concerns among MSM in several Asian settings (2). HIV testing supports early diagnosis, treatment, counseling, and linkage to prevention services (3). These services may include assessment for pre-exposure or post-exposure prophylaxis when appropriate (4).

The quality of testing services depends on more than laboratory procedures. Trust, respectful communication, and suitable outreach can affect whether MSM use testing services and remain engaged with them (5). Digital tools may support this work by collecting structured information before or during a testing encounter. Evidence from eHealth research suggests that these tools can assist HIV prevention, testing, and management, although their effects have varied across studies (6).

Sentinel surveillance programs routinely collect demographic and behavioral information through standard questionnaires. Most of these data are available before the current HIV result is known. Previous studies have used questionnaire data to estimate HIV risk among MSM. One Chinese study developed a risk model using demographic, behavioral, and psychosocial information (7). Another study compared machine-learning methods using MSM sentinel surveillance data and evaluated the models in a later year (8). A web-based model also showed that self-reported information could help predict HIV and other sexually transmitted infections (9).

However, good performance in development data does not ensure similar performance in a later period or another city. Questionnaire revisions, coding changes, prevention uptake, and changes in the population seeking testing may affect model performance (10). Independent validation is therefore needed before a model is considered for service use (11). Validation should also examine calibration and prediction error. A model may rank encounters well but still produce probabilities that do not match the observed risk (12).

We developed a questionnaire-based model to help prioritize optional additional services during HIV testing encounters among MSM. The model was not designed to determine who should receive HIV testing or routine services. All people should continue to receive standard testing, counseling, prevention services, and clinical care according to applicable procedures. Nantong records from 2020–2023 were used for model development, and records from 2024–2025 were reserved for temporal validation. The locked model was then evaluated in a separate geographic cohort from an external city in Shandong Province. We assessed discrimination, prediction error, calibration, performance at a locked threshold, and event capture under fixed workload levels.

## 2 Materials and methods

### 2.1 Study design and reporting

We conducted a retrospective prediction modeling study using de-identified MSM sentinel surveillance records. The model was developed using records from an earlier period and evaluated in two separate settings. The first validation cohort contained later records from the development city. The second contained surveillance records from an external city in Shandong Province.

Reporting followed the TRIPOD+AI statement (13). We also considered the main risk-of-bias and applicability domains in PROBAST+AI (14). Figure 1 shows cohort construction and the separation between model development, temporal validation, and geographic validation.

**Figure 1:**
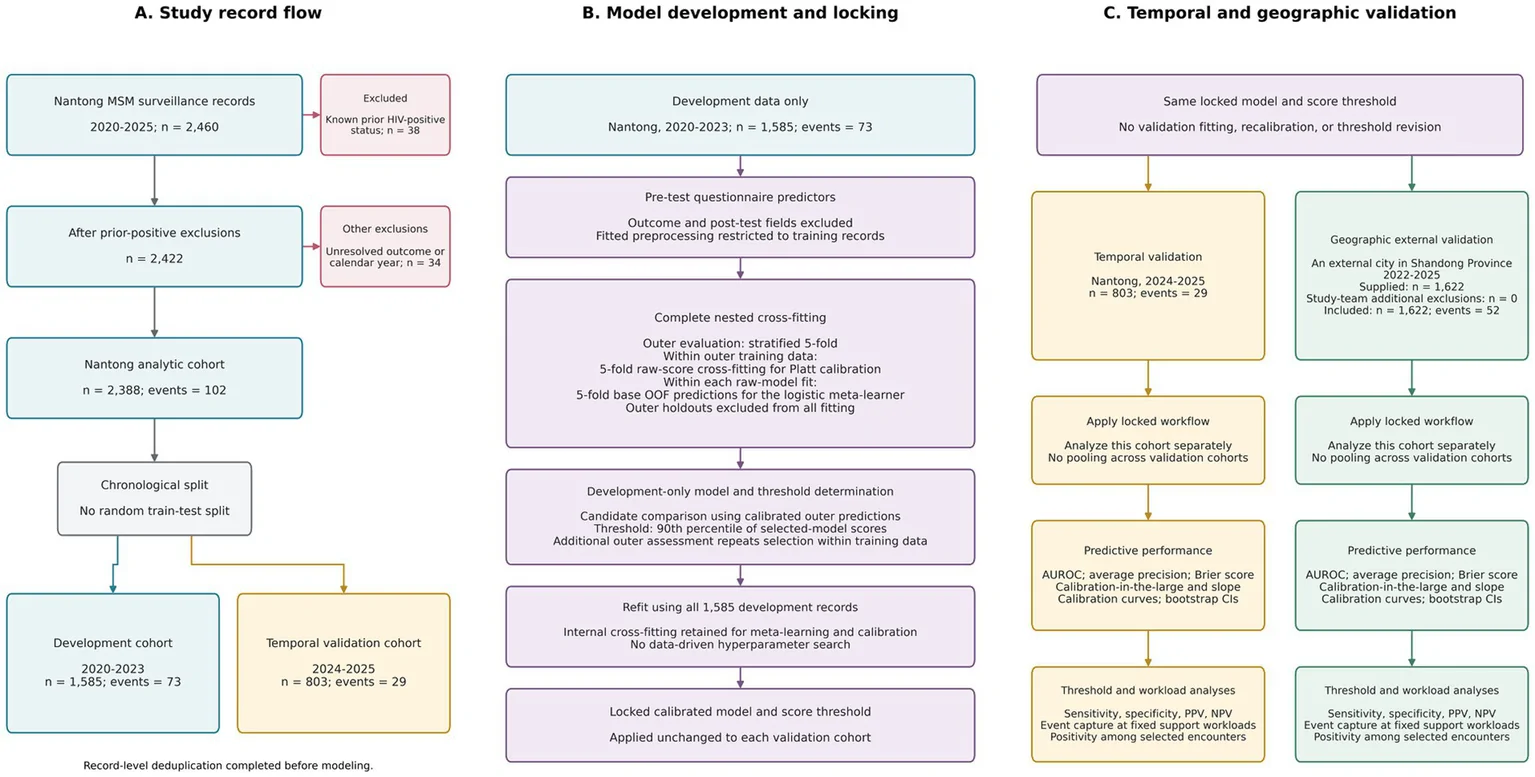
Study record flow, model development and locking, and temporal and geographic validation. Nantong records from 2020–2023 were used for model development, and records from 2024–2025 were reserved for temporal validation. The geographic external validation cohort contained records from 2022–2025 from an external city in Shandong Province. The study team included all 1,622 supplied records without further exclusions. These counts describe the received analytic file, not the provider’s full source population or upstream exclusions. Years were combined within each validation cohort, but the temporal and geographic cohorts were analyzed separately. Before modeling, disease control staff retained only the first recorded testing encounter for each person during the relevant study period. Nantong records were deduplicated across 2020–2025 before the temporal split, so no participant appeared in both the development and temporal validation cohorts. The external provider applied the same first-record rule across 2022–2025. In the revised analysis, all fitted preprocessing, model fitting, probability calibration, model selection, and threshold selection used development data only. Both validation cohorts had already been examined in the original analysis. The locked calibrated model and score threshold were then applied without refitting, recalibration, or adjustment in both validation cohorts. The score was evaluated as a tool for prioritizing optional additional support and was not used to restrict HIV testing, laboratory confirmation, routine counseling, prevention services, or clinical care. MSM, men who have sex with men.

### 2.2 Data sources and study population

The Nantong source dataset contained 2,460 testing encounters recorded from 2020 to 2025. We excluded 38 encounters involving people with a known previous HIV-positive status. A further 34 encounters were excluded because the current outcome or calendar year could not be resolved. The final Nantong dataset contained 2,388 encounters and 102 confirmed HIV-positive events.

Records from 2020–2023 formed the development cohort. It included 1,585 encounters and 73 confirmed events. Records from 2024–2025 formed the temporal validation cohort. It included 803 encounters and 29 confirmed events. The supplied geographic external dataset contained 1,622 encounters from 2022–2025, including 52 confirmed events. We included all supplied records without further exclusions by the study team. These counts describe the received dataset, not the provider’s full source population. We did not apply an additional exclusion for previously known HIV-positive status to the external file. All supplied records had a resolved binary outcome under the extraction rules described below. We combined years within each validation cohort but analyzed the temporal and geographic cohorts separately. They were not used to define preprocessing, fit or select a model, calibrate predictions, or determine the threshold. Cohort construction and analytical roles are detailed in Supplementary Table S1.

Before modeling, disease control staff identified records belonging to the same person and retained only the first recorded testing encounter during the study period. In Nantong, this process covered 2020–2025 and preceded the split into development and temporal validation cohorts. Each person therefore contributed one record and could not appear in both Nantong cohorts. The external data provider applied the same first-record rule across 2022–2025 before supplying the geographic validation dataset. Each analytic record represented one participant at the retained testing encounter. The primary analysis therefore already used first-record data. Here, “first” refers to the first recorded encounter during the study period, not necessarily the person’s first lifetime HIV test. Study size was based on all eligible records available during the study period.

### 2.3 Outcome and predictors

The outcome was a confirmed HIV-positive event during the same testing encounter. The recorded HIV antibody result represented the final result after completion of the applicable testing process. A confirmatory-positive result was coded as an event. A screening-negative result was coded as a non-event. A screening-positive result alone was not coded as an event unless confirmatory evidence was available. In Nantong, encounters with a recorded previous HIV-positive status were excluded. For the external dataset, the extraction procedure prioritized an explicit binary outcome field, followed by a confirmatory-result field and then the final recorded antibody-result field. These rules identified the source of the recorded outcome; they did not change the clinical event definition stated above. Section 2.2 describes the study-team exclusions for the external dataset. The outcome rules and checks are reported in Supplementary Table S2.

Candidate predictors were limited to information recorded before the current test result was known. They included age, marital status, education, testing history, previous sexually transmitted infections, sexual behaviour, condom use, and prevention-related information. Test results, post-test referrals, linkage records, follow-up information, direct identifiers, and administrative numbers were excluded.

We used a predefined feature list to reduce the risk of outcome leakage. D03 was retained because the available documentation supported its use as a pre-test questionnaire item. D04 was excluded from the primary model because its coding pattern suggested that it might act as a close proxy for the outcome. Supplementary Table S3 lists all predictors used in the primary model, including their English item wording, response options, database coding, questionnaire-version information, and model handling. It also lists the feature sets used in the sensitivity analyses.

### 2.4 Data preparation

We retained the original predictor definitions and database coding rules. Before imputation, recoding, or one-hot encoding, we counted the raw missing values and summarized the observed predictor distributions separately in the development, temporal-validation, and geographic-validation cohorts. We distinguished an item that was unavailable because it had not yet been introduced from a blank response in an available questionnaire. Where the source data provided a separate code, unknown and unclassified responses were retained as separate categories. Refusal could not be identified as a separate category because it was not recorded independently in the supplied data. Within cross-fitting, all preprocessing parameters were estimated using only the training records available at that level. The held-out records did not contribute to fitting the preprocessing used for their predictions. Numeric missing values were replaced with medians estimated from the relevant development fold. Blank categorical values were retained as a separate missing category, distinct from explicitly coded unknown responses. Categorical variables were one-hot encoded using categories learned from the relevant training data. Categories not observed during training were encoded as zeros across the corresponding item’s one-hot columns.

The questionnaire and its implementation differed in some respects across the study period and data sources. We therefore reviewed the item wording, response options, database coding, item availability, missingness, and annual distributions of each primary predictor. We documented the original coding and preprocessing rules for each predictor and reported differences in questionnaire wording and implementation across sources and years. This reporting audit did not change the model inputs or the locked preprocessing rules. Validation outcomes were not used to revise any predictor or preprocessing rule. Supplementary Table S3 reports the wording, response options, coding, version information, and model handling for all primary predictors. Supplementary Table S4 reports predictor availability, raw missing counts and percentages, and observed predictor distributions in the development, temporal-validation, and geographic-validation cohorts.

The raw missingness audit showed different availability patterns across cohorts. C09 was first implemented in 2022. In the development cohort, 784 of 1,585 records (49.5%) had no C09 value; all were from 2020–2021, before the item was introduced. C09 had no blank values in the temporal-validation cohort (0/803, 0%). In the geographic-validation cohort, C09 was available from 2022 onward, but completion was not mandatory in the local data collection system. In this cohort, 504 of 1,622 records (31.1%) had no recorded C09 response. These blanks were counted as raw missing values and reported separately from explicit “unsure” responses.

D03 was coded as a binary pre-test questionnaire item. Its raw blank counts were 96 of 1,585 records (6.1%) in the development cohort, 67 of 803 (8.3%) in the temporal-validation cohort, and 92 of 1,622 (5.7%) in the geographic-validation cohort. The blank pattern was consistent with the D01-based skip instructions in the available questionnaire documentation, but the reason for each blank was not independently recorded. We therefore report these values as raw blanks before model preprocessing. Complete missing counts, percentages, and observed distributions for all primary predictors are provided in Supplementary Table S4.

### 2.5 Model development and selection

The locked stacked ensemble combined penalized logistic regression, random forest, and HistGradientBoosting base learners through a logistic-regression meta-learner. The primary comparison retained the original two candidates: the calibrated stacked ensemble and HistGradientBoosting. We also evaluated penalized logistic regression and random forest as stand-alone models and applied the same selection tolerances in an exploratory four-model comparison. TabNet was examined during preliminary development as an exploratory tabular deep-learning method (15), but it was not included in the locked stack or the final model comparison.

We used stratified five-fold outer cross-fitting to evaluate each complete candidate workflow. Within each outer training set, a second five-fold procedure generated held-out raw scores for fitting Platt calibration. For the stack, each raw-model fit used a further five-fold procedure to generate base-model out-of-fold predictions. The logistic-regression meta-learner was fitted to these predictions. The base learners were then refitted on the relevant training records. The outer holdout was excluded from fitting the preprocessing, base learners, meta-learner, and calibrator. Each development record therefore received a calibrated prediction from a complete workflow fitted without that record. We retained the original base-model settings and performed no hyperparameter search in the revised analysis. The logistic meta-learner combined the base-model predictions, but this did not ensure calibration on new records. We retained a separate Platt step to adjust the final raw score and evaluated that step within the outer cross-fitting procedure. The calibrator used the logit of the raw score as its single predictor. It used logistic regression with weak L2 regularization (C = 1,000,000). This step was intended to improve calibration but did not guarantee accurate probabilities in the validation cohorts.

Candidate models were compared using the area under the receiver operating characteristic curve (AUROC), area under the precision–recall curve (AUPRC), Brier score, and Brier skill score. The selection rule first retained candidates within 0.01 of the highest calibrated development AUROC. It then retained candidates within 0.001 of the lowest Brier score among those candidates. HistGradientBoosting was preferred to the stack if both remained eligible. The exploratory four-model comparison used the same tolerances, with the preference order of penalized logistic regression, HistGradientBoosting, random forest, and the stack. AUPRC and Brier skill score were reported but were not additional selection steps. Paired bootstrap samples were used for model comparisons. In a separate assessment, we repeated the primary model-selection procedure within each outer training set and evaluated the selected workflow on the corresponding outer holdout.

### 2.6 Validation and statistical analysis

The locked model was applied separately to the temporal and geographic validation cohorts. Discrimination was assessed with AUROC. We also reported AUPRC using average precision because confirmed events were uncommon. Precision–recall measures can be useful when outcomes are imbalanced (16). Overall prediction error was assessed with the Brier score and Brier skill score. The Brier skill score used a constant prediction equal to the event prevalence in the relevant cohort as its reference. We estimated calibration-in-the-large with the score logit as an offset and its coefficient fixed at 1. We estimated the calibration slope in a separate logistic model with a freely estimated intercept. Ideal values are 0 for calibration-in-the-large and 1 for the slope. These models assessed calibration and did not update the predictions. Calibration plots used five approximately equal-sized groups ordered by score. We reported the number of encounters and events in each group, with Wilson 95% confidence intervals for the observed event proportions. Descriptive centered moving-window curves used 41 encounters in temporal validation and 81 in geographic validation. These curves did not recalibrate the model.

The operating threshold was defined as the 90th percentile of the selected model’s calibrated outer cross-fitted development scores, using linear interpolation. This produced a locked threshold of 0.09438. We selected encounters with scores at or above this threshold and applied it unchanged in both validation cohorts. Sensitivity, specificity, positive predictive value, negative predictive value, and the selected proportion were calculated.

We also examined the highest-scoring 5%, 10%, 20%, and 30% of encounters. We rounded the selected number upward and resolved score ties using the original record order. At each workload level, we calculated the number and proportion of events captured and the observed positivity among selected encounters. These descriptive analyses concerned optional additional support, not eligibility for HIV testing. They were separate from evaluation at the locked numeric threshold. Formal decision-curve analysis was not used as evidence of operational value because the benefits and harms of optional additional support had not been measured prospectively.

We compared missingness, category frequencies, predictor distributions, and score distributions across periods and study settings. We also reported annual descriptive performance. Within each cohort, we summarized scores by age group (<25, 25–34, 35–44, and ≥45 years) and recorded education category. Summaries included the mean, median, and 10th and 90th percentiles. Development summaries used outer cross-fitted scores, and validation summaries used locked-model scores. These analyses described demographic score patterns but did not establish equal predictive performance or fairness across groups.

We calculated percentile 95% confidence intervals from 1,000 bootstrap samples, with encounters resampled within outcome groups. Predictions remained fixed during resampling. Paired comparisons used the same sampled encounters for both models. These intervals describe uncertainty conditional on the saved predictions. They do not include uncertainty from refitting the full workflow. Each participant contributed one record to the analysis. The revised analysis used Python 3.10.21 and scikit-learn 1.7.2, with a master random seed of 42 and deterministic fold-specific seeds. Supplementary Table S16 reports the retained environment metadata.

### 2.7 Ethics

The Ethics Committee of the Nantong Center for Disease Control and Prevention approved this retrospective analysis of de-identified records (approval no. 2026–011; 15 May 2026). The external data were used under institutional authorization, applicable ethics arrangements, and an inter-institutional data-use agreement. The external city and data provider are therefore not identified.

The requirement for additional consent was waived because only previously collected, de-identified records were analyzed. No score changed testing, counseling, or care. The model was evaluated only as a possible service-support tool.

## 3 Results

### 3.1 Study cohorts and model selection

The final Nantong dataset contained 2,388 encounters and 102 confirmed events. The development cohort included 1,585 encounters and 73 events, with an event prevalence of 4.6%. Temporal validation included 803 encounters and 29 events, with a prevalence of 3.6%. Geographic external validation included 1,622 encounters and 52 events, with a prevalence of 3.2%. Table 1 presents the cohort characteristics and analytical roles. All candidate models were developed using the 2020–2023 cohort. The stacked ensemble was selected in the primary comparison with HistGradientBoosting using the development-only rule described in Section 2.5. With the complete calibrated workflow evaluated in outer held-out folds, the stack had an AUROC of 0.855, an AUPRC of 0.416, a Brier score of 0.0331, and a Brier skill score of 0.247. The corresponding HistGradientBoosting results were 0.841, 0.368, 0.0357, and 0.187. Penalized logistic regression alone had corresponding values of 0.826, 0.390, 0.0343, and 0.219. Paired differences for the stack minus HistGradientBoosting were 0.0142 for AUROC (95% CI −0.0062 to 0.0342), 0.0479 for AUPRC (0.0047 to 0.0963), and −0.00264 for the Brier score (−0.00427 to −0.00107). In the expanded comparison, random forest had a higher development AUROC than the stack (difference 0.024; 95% CI 0.009 to 0.042). It was selected when the same rule was applied to all four candidates. This additional comparison did not replace the primary model. The separate outer assessment that repeated primary model selection within each training fold selected the stack in all five folds. Its outer-held-out discrimination, prediction error, and calibration estimates matched the selected-stack estimates in Table 2 to the reported precision. This assessment evaluated the stated two-model selection procedure, not the earlier exploratory choices. Supplementary Tables S6 and S7 report separate validation and cross-fitted development results, respectively. Supplementary Table S5, Part E, reports absolute candidate-model performance, including calibration estimates and confidence intervals. Supplementary Table S18 reports paired model comparisons. In outer cross-fitted development predictions, the stack’s calibration-in-the-large changed from −0.167 before Platt calibration to −0.022 after calibration, and its calibration slope changed from 1.153 to 0.933. The Brier-score difference for calibrated minus raw scores was −0.00021 (95% CI −0.00093 to 0.00053). The point estimates moved closer to the ideal calibration values, but the Brier-score interval included zero. Neither validation cohort was combined with the other for performance estimation.

**Table 1:** Cohort characteristics and analytical roles of the development, temporal validation, and geographic external validation cohorts.

Characteristic	Development cohort	Temporal validation cohort	Geographic external validation cohort
Data source	MSM sentinel surveillance program in Nantong	MSM sentinel surveillance program in Nantong	Surveillance program in an external city in Shandong Province
Study period	2020–2023	2024–2025	2022–2025
Analytical role	Model development, selection, calibration, and threshold determination	Temporal validation	Geographic external validation
Testing encounters, n	1,585	803	1,622
Confirmed HIV-positive events, n (%)	73 (4.6)	29 (3.6)	52 (3.2)
Non-events, n (%)	1,512 (95.4)	774 (96.4)	1,570 (96.8)
Unit of analysis	One testing encounter per participant	One testing encounter per participant	One testing encounter per participant
Used to establish preprocessing rules	Yes	No	No
Used for model fitting	Yes	No	No
Used for model selection	Yes	No	No
Used for probability calibration	Yes	No	No
Used for threshold determination	Yes	No	No

Values are *n* (%) unless otherwise stated. Each participant contributed one HIV testing encounter: the first recorded encounter during the relevant study period. The outcome was a confirmed HIV-positive event during that encounter. Disease control staff completed person-level deduplication before modeling. Nantong deduplication covered 2020–2025 before the temporal split, so no participant appeared in both Nantong cohorts. The external provider applied the same first-record rule across 2022–2025. The external cohort included all 1,622 supplied records, with no further exclusions by the study team. These counts describe the received analytic file, not the provider’s full source population. Section 2.2 describes the external inclusion rules. The external cohort was not used to fit preprocessing, select or fit the model, calibrate predictions, or determine the threshold. MSM, men who have sex with men.

**Table 2:** Cross-fitted development performance and locked-model performance in the temporal and geographic validation cohorts.

Performance measure	Development cohort, 2020–2023, cross-fitted	Temporal validation, 2024–2025	Geographic external validation, 2022–2025
Testing encounters, *n*	1,585	803	1,622
Confirmed HIV-positive events, *n*	73	29	52
Event prevalence, %	4.6	3.6	3.2
AUROC	0.855 (0.798–0.902)	0.963 (0.930–0.990)	0.974 (0.955–0.989)
AUPRC	0.416 (0.312–0.548)	0.696 (0.536–0.880)	0.746 (0.652–0.842)
Brier score	0.0331 (0.0284–0.0375)	0.0165 (0.0119–0.0215)	0.0153 (0.0120–0.0186)
Brier skill score	0.247 (0.147–0.354)	0.527 (0.382–0.659)	0.507 (0.402–0.613)
Calibration-in-the-large	−0.022 (−0.175 to 0.121)	−0.118 (−0.338 to 0.089)	−0.357 (−0.523 to −0.205)
Calibration slope	0.933 (0.783–1.102)	1.530 (1.206–2.064)	1.414 (1.245–1.663)
Prediction source	Cross-fitted out-of-fold predictions from the selected model	Locked-model predictions	Locked-model predictions
Validation data used for refitting or recalibration	Not applicable	No	No

Values in parentheses are percentile 95% confidence intervals from 1,000 stratified encounter-level bootstrap samples of fixed predictions. Development estimates used calibrated outer cross-fitted predictions for the selected architecture. They do not represent a fully selection-adjusted estimate of the model-selection procedure. Validation estimates used the locked calibrated stack without refitting or recalibration. AUPRC was summarized using average precision. Calibration-in-the-large was estimated with the score logit as an offset and its coefficient fixed at 1. The calibration slope was estimated in a separate logistic model with a freely estimated intercept. Ideal values are 0 and 1, respectively. These models assessed calibration and did not update predictions. Bootstrap intervals do not include uncertainty from refitting the full modeling workflow. AUROC, area under the receiver operating characteristic curve; AUPRC, area under the precision–recall curve.

### 3.2 Validation performance

The locked model had AUROC values above 0.95 in both validation cohorts. Temporal-validation AUROC was 0.963 (95% confidence interval [CI] 0.930–0.990), and AUPRC was 0.696 (95% CI 0.536–0.880). Geographic-validation AUROC was 0.974 (95% CI 0.955–0.989), and AUPRC was 0.746 (95% CI 0.652–0.842). The Brier scores were 0.0165 and 0.0153, respectively. Calibration-in-the-large was −0.118 (95% CI −0.338 to 0.089) in temporal validation and −0.357 (−0.523 to −0.205) in geographic validation. The corresponding calibration slopes were 1.530 (1.206–2.064) and 1.414 (1.245–1.663). Full performance estimates are presented in Table 2. Discrimination, calibration, and fixed-workload performance are shown in Figure 2. The calibration panel includes group sizes, event counts, Wilson intervals, and descriptive moving-window curves. The corresponding bootstrap confidence intervals and calibration estimates are provided in Supplementary Table S8.

**Figure 2:**
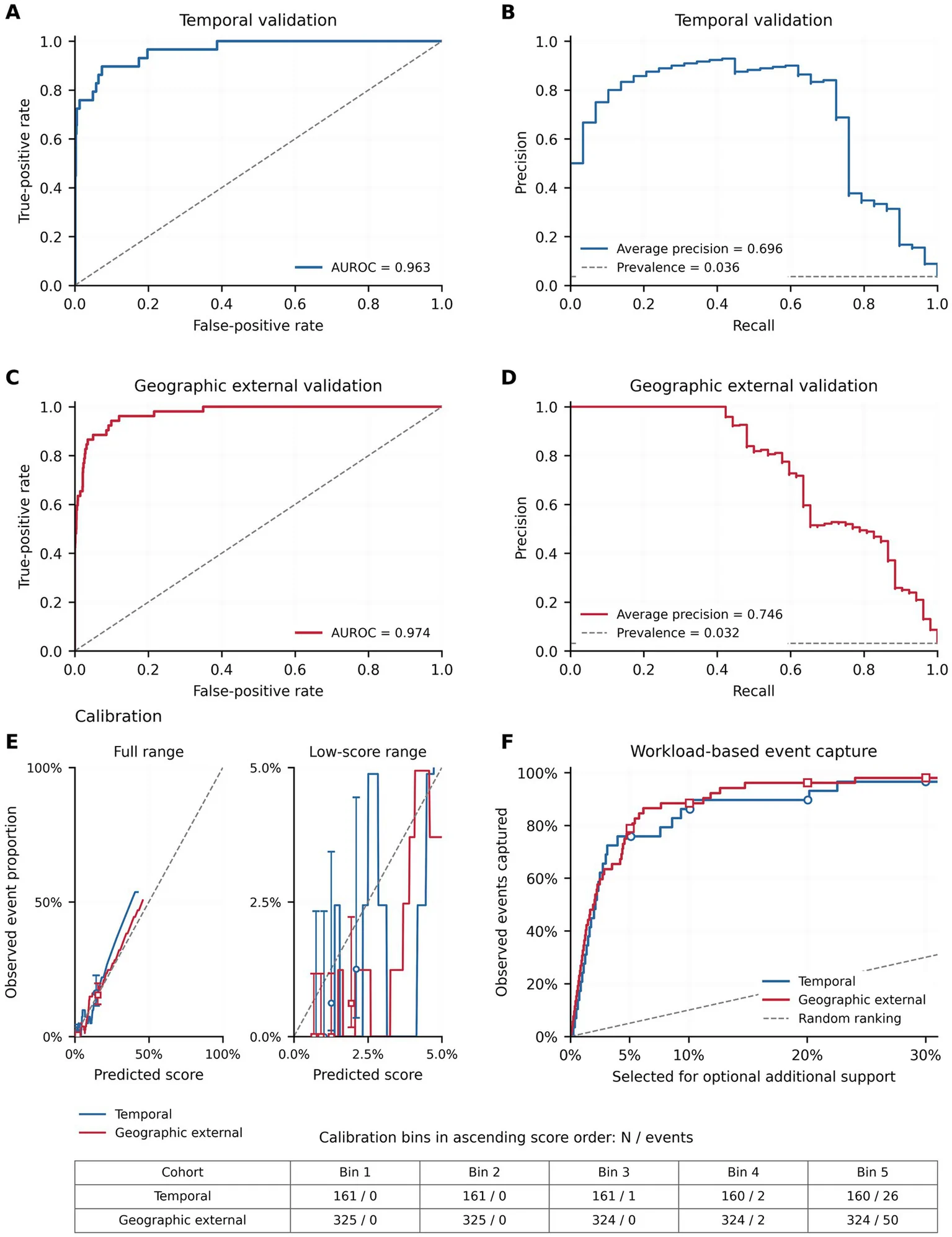
Discrimination, calibration, and workload-based event capture in the temporal and geographic validation cohorts. Panels **A** and **B** show the receiver operating characteristic and precision–recall curves in temporal validation. Panels **C** and **D** show the corresponding curves in geographic external validation. Average precision was used to summarize precision–recall performance. The dashed horizontal lines in Panels **B** and **D** show the observed event prevalence in the corresponding cohort. Panel **E** shows calibration over the full score range and within the low-score range. The markers represent five approximately equal-sized groups ordered by predicted score, and the error bars indicate Wilson 95% confidence intervals for the observed event proportions. The solid lines are descriptive centered moving-window estimates, using 41 records in the temporal cohort and 81 records in the geographic external-validation cohort. These lines do not represent model recalibration or confidence bands. The table below the panel reports the number of encounters and confirmed events in each calibration group. Panel **F** shows the cumulative proportion of confirmed events captured when encounters are ranked from the highest to the lowest locked-model score for optional additional support. Markers indicate nominal workload levels of 5%, 10%, 20%, and 30%, with selected counts rounded up to the next whole encounter. The diagonal dashed line represents expected event capture under random selection. This fixed-workload analysis was descriptive and did not change the locked model or score threshold. It does not restrict HIV testing, laboratory confirmation, routine counseling, prevention services, or clinical care.

### 3.3 Locked-threshold and workload performance

At the locked threshold of 0.0944, the model selected 45 of 803 temporal-validation encounters (5.6%). It identified 22 of 29 events, giving 75.9% sensitivity, 97.0% specificity, and 48.9% positive predictive value. In geographic validation, it selected 103 of 1,622 encounters (6.4%) and identified 45 of 52 events. Sensitivity was 86.5%, specificity was 96.3%, and positive predictive value was 43.7%. Seven events fell below the threshold in each cohort. Table 3 presents the threshold and fixed-workload results.

**Table 3:** Performance at the development-locked score threshold and under fixed workload levels for optional additional support.

Performance measure	Temporal validation, 2024–2025	Geographic external validation, 2022–2025
Panel A. Performance at the development-locked score threshold of 0.0944 for optional additional support		
Total testing encounters, *n*	803	1,622
Confirmed HIV-positive events, *n*	29	52
Encounters prioritized for optional additional support, *n* (%)	45 (5.6)	103 (6.4)
True positives, *n*	22	45
False positives, *n*	23	58
False negatives, *n*	7	7
True negatives, *n*	751	1,512
Sensitivity, %	75.9	86.5
Specificity, %	97.0	96.3
Positive predictive value, %	48.9	43.7
Negative predictive value, %	99.1	99.5

Validation cohort	Nominal workload	Encounters prioritized, *n*	Confirmed events among prioritized encounters, *n*/*N* (%)	Observed event proportion among prioritized encounters, %	Prioritized encounters per captured event
Panel B. Event capture when encounters are ranked for optional additional support					
Temporal validation	5%	41	22/29 (75.9)	53.7	1.86
Temporal validation	10%	81	25/29 (86.2)	30.9	3.24
Temporal validation	20%	161	26/29 (89.7)	16.1	6.19
Temporal validation	30%	241	28/29 (96.6)	11.6	8.61
Geographic external validation	5%	82	41/52 (78.8)	50.0	2.00
Geographic external validation	10%	163	46/52 (88.5)	28.2	3.54
Geographic external validation	20%	325	50/52 (96.2)	15.4	6.50
Geographic external validation	30%	487	51/52 (98.1)	10.5	9.55

The operating threshold was the 90th percentile of the selected model’s calibrated outer cross-fitted development scores, calculated using linear interpolation. The exact locked value was 0.09438076500258265, reported here as 0.0944. The threshold was applied unchanged in both validation cohorts. Fixed-workload analyses ranked encounters from the highest to the lowest locked-model score. At each nominal workload, the number prioritized was calculated by multiplying the cohort size by the workload proportion and rounding up to the next whole encounter. Score ties were resolved using the original record order. These analyses were descriptive and were not used for model fitting, model selection, probability calibration, or threshold selection. The analyses describe the possible prioritization of encounters for optional additional support. They do not restrict HIV testing, laboratory confirmation, routine counseling, prevention services, or clinical care.

The full confusion matrices and threshold results are reported in Supplementary Table S9. At the nominal 10% workload level, the model selected 81 temporal-validation encounters and 163 geographic-validation encounters. It captured 25 of 29 and 46 of 52 events, respectively. These values corresponded to event-capture rates of 86.2 and 88.5%. At the 20% workload level, event capture increased to 89.7% (26/29) and 96.2% (50/52), respectively. These ranking-based analyses used fixed selection fractions rather than the locked numerical threshold. Complete workload results are provided in Supplementary Table S10. The reasons for not using formal decision-curve analysis as evidence of operational benefit are set out in Supplementary Table S11.

### 3.4 Distribution shift and sensitivity analyses

Predictor distributions differed across periods and study settings. The differences involved questionnaire availability, coding, missingness, and category frequencies. C09 illustrates this distinction. In the development cohort, the 784 blank C09 values were concentrated in 2020–2021, before the item was introduced. In the geographic-validation cohort, completion of C09 was not mandatory in the local data collection system, and 504 records from 2022 to 2025 had no recorded response. These two types of blank values were represented in the model as missing input values, but they arose from different questionnaire or implementation conditions. The model was not updated in response because validation was intended to assess the locked workflow. The main distribution differences are summarized in Supplementary Table S12, and complete predictor-level counts and distributions are reported in Supplementary Table S4. Record-level scores were retained in the controlled institutional archive for metric calculation and audit, as described in Supplementary Table S13. Score distributions also differed by age and education (Supplementary Table S17). Encounters among people aged 45 years or older had the highest mean scores in all three cohorts. In temporal and geographic validation, their mean scores were 0.144 and 0.114, compared with 0.024 and 0.022 among those younger than 25 years. Median scores in these age groups were 0.026 versus 0.012 and 0.027 versus 0.011, respectively. Across the observed education groups, median scores ranged from 0.009 to 0.048 in temporal validation and from 0.012 to 0.034 in geographic validation. These descriptive differences do not establish either fairness or bias, especially where groups contained few encounters or events.

Removing C09 reduced AUROC from 0.963 to 0.932 in temporal validation and from 0.974 to 0.949 in geographic validation. Removing D03 produced larger reductions, with AUROC values of 0.890 and 0.939. The paired AUROC differences after removing D03 were −0.073 (95% CI −0.129 to −0.026) and −0.035 (−0.064 to −0.010), respectively. Removing both D02 and D03 yielded AUROC values of 0.865 and 0.927. These comparisons describe the contribution of the variables to the locked modeling workflow. They do not establish causal effects, and the change after removing C09 may also reflect the different availability and missingness patterns of this item across cohorts. Adding D04 increased geographic-validation AUROC to 0.996 and AUPRC to 0.916, but D04 remained excluded because of concern that it could act as an outcome proxy. Full sensitivity results and paired differences are reported in Supplementary Table S14. Numerical consistency and model-locking checks are reported in Supplementary Table S15. The software environment and reproducibility settings are reported in Supplementary Table S16.

## 4 Discussion

### 4.1 Principal findings

This study developed a questionnaire-based model to rank MSM testing encounters for possible optional additional support. The model was developed with earlier Nantong records and assessed at the encounter level in later Nantong records and a separate geographic cohort. It showed good ranking performance in both settings and captured most confirmed events at the workload levels examined in this study.

The model is not a diagnostic test. Its main function is to rank encounters before the current result is known. At the locked threshold, it selected 5.6% of temporal-validation encounters and 6.4% of geographic-validation encounters. At this threshold, it identified 75.9 and 86.5% of confirmed events in the two validation cohorts. A nominal 10% workload captured 86.2 and 88.5% of events. These findings suggest that the score could help services plan optional counseling, prevention assessment, peer support, or follow-up. However, this retrospective study did not show whether score-guided support improves service or health outcomes.

### 4.2 Comparison with previous work

Earlier studies found that demographic and behavioral questionnaires could support HIV risk assessment among MSM. However, many models were assessed mainly by internal data splitting or within a single surveillance system. Our study used both later records from the development city and a separate geographic cohort. In the revised analysis, all fitting and selection steps used development data only, and the resulting workflow was applied unchanged to each validation cohort. However, these cohorts had already been examined in the original analysis. They are not newly untouched validation datasets.

A recent systematic review found that machine-learning models for HIV and other sexually transmitted infections often reported good discrimination. However, methods and results varied, and external validation remained uncommon (17). An independent evaluation of the MySTIRisk tool found lower discrimination than in its original study, although the model retained useful predictive ability (18). These findings show why performance should be tested in a new setting without local refitting.

The validation estimates were higher than the cross-fitted development results. The development estimates came from held-out records, whereas validation used the model refitted on all development records. Differences in case mix, questionnaire coding, predictor distributions, and sampling variation may also contribute. Annual development AUROC estimates ranged from 0.626 in 2020 to 0.995 in 2023 (Supplementary Table S12). Each year contained only 12–27 events. These descriptive differences show that performance varied within the development period, but they do not establish the cause of the development–validation gap. The stack had a higher development AUROC than penalized logistic regression, but the paired AUPRC and Brier-score intervals included no difference. Random forest had a higher development AUROC than the stack in the expanded comparison. These findings do not establish that the stack is the best model for every setting or that its added complexity will improve service outcomes. They also do not mean that performance will always improve in new settings. Temporal dataset shift can affect a model in either direction (19). Validation in other regions remains necessary.

### 4.3 Interpretation and monitoring

Workload-based results may be easier for services to interpret than a single probability threshold. Services differ in staff numbers and available support. They may therefore need to consider how many encounters can receive additional attention. Still, a low score must never reduce standard care. Events remained among encounters below the threshold. The score must not be used to deny testing, delay confirmation, or restrict prevention services.

Calibration also requires caution. Calibration-in-the-large was negative in both validation cohorts, although its interval included zero in temporal validation. The geographic-validation result indicated overall overprediction. Both calibration slopes were above 1, with intervals excluding 1. This suggests that predicted log-odds varied less than the observed outcome pattern required. A low Brier score therefore did not establish accurate individual probabilities. Staff should use the scores for relative ranking and should not communicate them as personal HIV-risk percentages. Training and any future interface should make this distinction clear.

Some predictor distributions changed over time and between cities. These changes may reflect questionnaire revisions, coding practice, or differences in people seeking testing. Services should monitor missingness, category frequencies, score distributions, workload, event capture, and calibration. A medical algorithm audit may support this process by reviewing data quality, model performance, workflow, and governance together (20).

Sensitivity analyses showed dependence on C09 and D03, with further changes when D02 and D03 were removed together. These findings do not imply that the variables have causal effects. Before use elsewhere, services should check the wording, coding, timing, and availability of these items. A model that relies on them may perform differently if another service records them in a different way. D04 improved some measures, but improved performance alone does not prove or rule out leakage. Its coding raised concern about a possible outcome proxy, so it remained excluded from the primary model.

### 4.4 Strengths and limitations

A main strength was the clear separation between model development, temporal validation, and geographic validation. The revised workflow kept all fitted preprocessing, model selection, calibration, and threshold selection within the development data. The outer evaluation excluded each held-out record from all fitting stages. The earlier inspection of validation results remains a limitation of this reanalysis. We examined discrimination, prediction error, calibration, performance at the locked threshold, fixed-workload performance, distribution shift, and dependence on selected predictors.

The study also had limitations. First, the event counts were modest. This reduced precision and did not support reliable subgroup or fairness analyses. Overall results do not establish equal performance across demographic or service groups. Algorithmic fairness requires direct assessment rather than inference from overall accuracy (21). Bias may also arise when an apparently neutral target reflects unequal access or service patterns (22). Second, the analysis included only the first recorded testing encounter for each participant during the study period. The findings therefore do not directly establish performance at later repeat-testing encounters. Bootstrap intervals condition on the saved predictions and do not capture uncertainty from refitting the full workflow. Development cross-fitted predictions share training data, which further limits interpretation of their bootstrap intervals. Third, questionnaire responses were self-reported and may have been affected by recall or social-desirability bias. Fourth, validation involved one external city, which limits geographic generalizability. The study team did not apply an additional exclusion for previously known HIV-positive status to the supplied external file. We therefore could not establish that this aspect of eligibility was identical across settings. Finally, the retrospective design did not assess staff use, acceptability to service users, service outcomes, or unintended effects.

### 4.5 Implications for research and practice

The next step should be prospective evaluation within the intended workflow. One possible design would begin with a 6–12-month silent phase at several services. Scores would be recorded but would not guide care. This phase would assess calibration, workload, missed-event patterns, and score distributions across age and education groups. It would also check whether staff understand that the score is not a personal HIV-risk probability. If performance and safety were acceptable, a subsequent cluster-randomized evaluation could compare score-guided optional support with usual support allocation over about 12 months. All groups would continue to receive standard testing and care. Outcomes could include uptake and completion of additional counseling, appropriate prevention assessment, linkage after a positive result, staff time, service-user acceptability, and unintended effects. These durations are planning examples, not an approved study protocol. A separate protocol should define sample size, event targets, subgroup assessments, and stopping rules before recruitment.

DECIDE-AI guidance recommends that early live evaluation examine human factors, workflow, safety, and unintended consequences (23). Governance should also address transparency, accountability, data protection, and inappropriate use (24). Good retrospective performance alone does not establish clinical or public-health benefit (25). Staff must retain professional judgment, and every service user must continue to receive standard testing and counseling.

### 4.6 Conclusion

Routine pre-test questionnaire data supported the ranking of HIV testing encounters in both temporal and geographic validation. The locked model captured most confirmed events at the workload levels examined, but the study did not establish that score-guided support improves service or health outcomes. Its scores are not exact individual probabilities and must not replace testing, laboratory confirmation, or professional judgment. Prospective evaluation, calibration monitoring, and fairness assessment are required before routine use.

## Patient and public involvement

Patients and members of the public were not involved in the design, conduct, analysis, interpretation, reporting, or dissemination planning of this retrospective study.

## Code availability

The analysis code may be considered for access upon reasonable request to the corresponding author, subject to institutional approval. Any shared materials would exclude record-level data, credentials, institution-specific file paths, and information that could identify individuals or the external data provider. Access to code would not imply access to either the Nantong or geographic external validation data.

## Data Availability

The data analyzed in this study is subject to the following licenses/restrictions: The individual-level datasets are not publicly available because they contain sensitive HIV-related and behavioral surveillance information and are subject to institutional authorization, applicable ethics arrangements, and inter-institutional data-use agreements. Only de-identified data were accessed for this study. The datasets cannot be shared openly or redistributed without approval from the relevant data-providing institutions and ethics authorities. Aggregated results may be available from the corresponding author upon reasonable request, subject to institutional and ethical review. Requests to access these datasets should be directed to xc6060@126.com.
