# Supplementary material for: A questionnaire-based model to prioritize additional support during HIV testing encounters among men who have sex with men: temporal and geographic external validation
Source: Front Public Health. 2026 Oct 16;14:1944057. doi: 10.3389/fpubh.2026.1944057 (PMC13720904; doi:10.3389/fpubh.2026.1944057)
Supplement: Supplementary file 1 [file Data_Sheet_1.docx]

# Supplementary Material

## Manuscript title

**A Questionnaire-Based Model to Prioritize Additional Support During HIV Testing Encounters Among Men Who Have Sex with Men: Temporal and Geographic External Validation**

## Contents

| **Item** | **Title** |
| --- | --- |
| Supplementary Table S1 | Cohort construction, aggregate counts, and analytical roles |
| Supplementary Table S2 | Outcome definition and quality-control audit |
| Supplementary Table S3 | Primary predictor definitions, response coding, questionnaire differences, and sensitivity feature sets |
| Supplementary Table S4 | Raw predictor missingness, distributions, and preprocessing in the three cohorts |
| Supplementary Table S5 | Model development, selection, calibration, and locking procedure |
| Supplementary Table S6 | Temporal and geographic validation performance of the locked model |
| Supplementary Table S7 | Cross-fitted development performance of the selected model |
| Supplementary Table S8 | Bootstrap confidence intervals and calibration estimates |
| Supplementary Table S9 | Performance at the development-locked threshold |
| Supplementary Table S10 | Fixed-workload analyses |
| Supplementary Table S11 | Rationale for the handling of decision-curve analysis |
| Supplementary Table S12 | Distribution differences and annual descriptive performance |
| Supplementary Table S13 | Validation prediction scores and data governance |
| Supplementary Table S14 | Sensitivity analyses |
| Supplementary Table S15 | Numerical consistency and model-locking checklist |
| Supplementary Table S16 | Software environment and reproducibility settings |
| Supplementary Table S17 | Score distributions by age group and education category |
| Supplementary Table S18 | Paired comparisons of candidate models |

**Supplementary Table S1. Cohort construction, aggregate counts, and analytical roles of the study datasets.**

## Part A. Cohort construction

| **Cohort or processing step** | **Study period** | **Records, n** | **Confirmed HIV-positive events, n** | **Non-events, n** | **Event prevalence, %** |
| --- | --- | --- | --- | --- | --- |
| Nantong source records | 2020–2025 | 2,460 | Not applicable | Not applicable | Not applicable |
| Previously known HIV-positive status excluded | 2020–2025 | 38 | Not applicable | Not applicable | Not applicable |
| Unresolved outcome or calendar year excluded | 2020–2025 | 34 | Not applicable | Not applicable | Not applicable |
| Final Nantong analytic dataset | 2020–2025 | 2,388 | 102 | 2,286 | 4.3 |
| Development cohort | 2020–2023 | 1,585 | 73 | 1,512 | 4.6 |
| Temporal validation cohort | 2024–2025 | 803 | 29 | 774 | 3.6 |
| Geographic external validation cohort | 2022–2025 | 1,622 | 52 | 1,570 | 3.2 |

## Part B. Annual counts in the Nantong analytic dataset

| **Year** | **Testing encounters, n** | **Confirmed HIV-positive events, n** | **Non-events, n** | **Event prevalence, %** |
| --- | --- | --- | --- | --- |
| 2020 | 395 | 27 | 368 | 6.8 |
| 2021 | 389 | 12 | 377 | 3.1 |
| 2022 | 392 | 16 | 376 | 4.1 |
| 2023 | 409 | 18 | 391 | 4.4 |
| 2024 | 409 | 17 | 392 | 4.2 |
| 2025 | 394 | 12 | 382 | 3.0 |
| Total | 2,388 | 102 | 2,286 | 4.3 |

## Part C. Analytical role of each cohort

| **Cohort** | **Model fitting** | **Preprocessing development** | **Probability calibration** | **Model selection** | **Threshold selection** | **Performance evaluation** |
| --- | --- | --- | --- | --- | --- | --- |
| Development cohort | Yes | Yes | Yes | Yes | Yes | Cross-fitted development estimates |
| Temporal validation cohort | No | No | No | No | No | Locked-model validation |
| Geographic external validation cohort | No | No | No | No | No | Locked-model validation |

**Note: Each participant contributed one HIV testing encounter: the first recorded encounter during the relevant study period. Disease control staff completed person-level deduplication before modeling. In Nantong, this process covered 2020–2025 before the development and temporal validation split. No participant therefore appeared in both Nantong cohorts. The external provider applied the same first-record rule across 2022–2025. The primary analysis already used these first-record datasets. The external file contained 1,622 records from 2022–2025, with 52 events and 1,570 non-events. The study team included all supplied records without further exclusions. These counts describe the received analytic file, not the provider’s full source population or upstream exclusions. Years were combined within each validation cohort, but the temporal and geographic cohorts were analyzed separately.**

**Supplementary Table S2. Outcome definition and quality-control audit.**

## Part A. Outcome definition

| **Item** | **Definition** |
| --- | --- |
| Primary outcome | Confirmed HIV-positive event during the same testing encounter |
| Event rule | A record with a confirmatory-positive HIV antibody result was coded as an event |
| Screening-negative rule | A screening-negative record was coded as a non-event |
| Screening-positive-only rule | A screening-positive result alone was not coded as an event unless confirmatory evidence was available |
| Previously diagnosed infection | In Nantong, encounters with recorded previous HIV-positive status were excluded. The study team did not apply an additional previous-positive exclusion to the supplied external file. |
| External outcome extraction | An explicit binary outcome field took priority, followed by a confirmatory-result field and then the final recorded antibody-result field. All 1,622 supplied records had a resolved binary outcome under these rules. |
| Prediction time point | Questionnaire information available before the current HIV test result was known |
| Unit of analysis | One HIV testing encounter per participant |
| Score direction | A higher score indicated higher priority for possible additional service support |
| Intended interpretation | Operational prioritization score rather than a diagnostic result or exact individual probability |

## Part B. Nantong quality-control audit

| **Audit item** | **Observed** | **Expected** | **Status** |
| --- | --- | --- | --- |
| Source records | 2,460 | 2,460 | Matched |
| Previously known HIV-positive exclusions | 38 | 38 | Matched |
| Unresolved outcome or calendar-year exclusions | 34 | 34 | Matched |
| Final analytic records | 2,388 | 2,388 | Matched |
| Total confirmed events | 102 | 102 | Matched |
| Development records | 1,585 | 1,585 | Matched |
| Development confirmed events | 73 | 73 | Matched |
| Temporal validation records | 803 | 803 | Matched |
| Temporal validation confirmed events | 29 | 29 | Matched |
| Both outcome classes present in development | Yes | Yes | Matched |
| Both outcome classes present in temporal validation | Yes | Yes | Matched |

## Part C. Geographic external validation audit

| **Audit item** | **Observed** | **Expected** | **Status** |
| --- | --- | --- | --- |
| Records supplied | 1,622 | 1,622 | Matched |
| Records included in analysis | 1,622 | 1,622 | Matched |
| Confirmed events | 52 | 52 | Matched |
| Non-events | 1,570 | 1,570 | Matched |
| Both outcome classes present | Yes | Yes | Matched |
| Used for preprocessing development | No | No | Matched |
| Used for model fitting | No | No | Matched |
| Used for probability calibration | No | No | Matched |
| Used for model selection | No | No | Matched |
| Used for threshold selection | No | No | Matched |

**Note:** The annual Nantong event counts were checked by the local HIV/AIDS and sexually transmitted infection control team before analysis. The geographic external validation data were analyzed separately and were not used to revise the outcome rule, predictor coding, model architecture, probability calibration, or operating threshold.

**Supplementary Table S3**. Primary predictor definitions, response coding, questionnaire differences, and sensitivity feature sets****

## Part A. English item wording and response coding for all 25 primary predictors

| **Variable** | **English item wording or field definition** | **Response options and database coding** | **Model input type** |
| --- | --- | --- | --- |
| age | Age in years, as recorded in the exported age field. | Numeric age in years. | Numeric |
| D02 | How many times have you had anal intercourse with male partners in the past week? | Reported number of acts; 0 is a valid recorded value. | Numeric |
| marital_status | Current marital status with a female partner. | 1 = Unmarried; 2 = Married; 3 = Cohabiting; 4 = Divorced or widowed. | Categorical |
| registered | Place of household registration. | 1 = Same province; 2 = Another province; 3 = Foreign nationality. | Categorical |
| educational_level | Educational attainment. | 1 = Illiterate; 2 = Primary school; 3 = Junior middle school; 4 = Senior middle school or technical secondary school; 5 = Junior college or above. | Categorical |
| B07 | Which of the following describes your sexual orientation? | 1 = Homosexual; 2 = Heterosexual; 3 = Bisexual; 4 = Undetermined. | Categorical |
| B08 | What is your main venue or method for finding male sexual partners? | 1 = Bar or dance hall; 2 = Tea house or club; 3 = Bathhouse; 4 = Park, public toilet, or outdoor grassy area; 5 = Internet or dating application; 6 = Other. | Categorical |
| C01 | Is AIDS an incurable, serious infectious disease? | 1 = Yes; 0 = No; 888 = Do not know. | Categorical |
| C02 | Are men who have sex with men currently the population most severely affected by AIDS in China? | 1 = Yes; 0 = No; 888 = Do not know. | Categorical |
| C03 | Can you tell whether a person has HIV by their appearance? | 1 = Yes; 0 = No; 888 = Do not know. | Categorical |
| C04 | Does having another sexually transmitted infection increase the risk of HIV infection? | 1 = Yes; 0 = No; 888 = Do not know. | Categorical |
| C05 | Can consistent and correct condom use reduce the risk of acquiring and transmitting HIV? | 1 = Yes; 0 = No; 888 = Do not know. | Categorical |
| C06 | Does using synthetic drugs, such as methamphetamine, ecstasy, or ketamine, increase the risk of HIV infection? | 1 = Yes; 0 = No; 888 = Do not know. | Categorical |
| C07 | Should people actively seek HIV testing and counseling after high-risk behavior, such as sharing injecting equipment or unprotected sex? | 1 = Yes; 0 = No; 888 = Do not know. | Categorical |
| C08 | Does deliberately transmitting HIV carry legal liability? | 1 = Yes; 0 = No; 888 = Do not know. | Categorical |
| C09 | How high do you think your risk of HIV infection is? | 1 = None; 2 = Low; 3 = Average or moderate, depending on questionnaire wording; 4 = High or very high; 5 = Unsure. See Part B for questionnaire and local implementation differences. | Categorical |
| D01 | Have you had anal intercourse with male partners in the past six months? | 1 = Yes; 0 = No. | Categorical |
| D03 | During the past six months, did you use a condom at your most recent anal intercourse with a male partner? | 1 = Yes; 0 = No. | Categorical |
| E01 | Have you had commercial sex with male partners in the past six months? | 1 = Yes; 0 = No. | Categorical |
| F01 | Have you had sex with female partners in the past six months? | 1 = Yes; 0 = No. | Categorical |
| G01 | Do you currently use drugs? | 1 = Yes; 0 = No. This was the locally implemented wording used in the study. | Categorical |
| H01 | Have you been diagnosed with a sexually transmitted infection in the past year? | 1 = Yes; 0 = No. | Categorical |
| I01 | In the past year, have you received condom promotion and distribution or HIV counseling and testing services? | 1 = Yes; 0 = No. | Categorical |
| I02 | In the past year, have you received community drug maintenance treatment or clean needle provision or exchange services? | 1 = Yes; 0 = No. | Categorical |
| I03 | In the past year, have you received peer education? | 1 = Yes; 0 = No. | Categorical |

## Part B. Questionnaire differences, local implementation, and handling in the original modeling workflow

| **Predictor or group** | **Questionnaire or implementation finding** | **Handling in the modeling workflow and reporting** |
| --- | --- | --- |
| age | Age was read from the exported age field. | The reporting audit did not recalculate age from birth year. Age remained numeric. |
| D02 | The available 2022 and 2025 questionnaires ask about the number of male anal intercourse acts in the past week. Both specify a D01-based skip to F01 when D01 = 0. | The original numeric values were retained. Blank values were treated as missing and imputed using the relevant training median. Recorded zeros were not recoded as missing. |
| marital_status; registered; B07; B08 | No wording or option difference was identified between the available 2022 and 2025 questionnaire items. | The exported categories were retained and one-hot encoded. The audit did not introduce a new category mapping. |
| educational_level | The study team confirmed that code 4 meant senior middle school or technical secondary school and code 5 meant junior college or above across study years and sources. | The original codes were retained. Differences in category frequencies were reported without exchanging or merging codes 4 and 5. |
| C01–C08 | The available 2022 and 2025 items showed no identified wording or coding difference. Database code 888 denotes an explicit “do not know” response. | The original response codes were retained as categories. Code 888 was kept separate from blanks. Responses were not converted into correctness scores. |
| C09: questionnaire wording | The 2022 questionnaire lists codes 1–4, with code 3 described as “average.” The 2025 questionnaire describes code 3 as “moderate” and also lists code 5, “unsure.” | Code 3 was retained as the same recorded category; no numeric risk scale was imposed. Code 5 was kept separate from both codes 1–4 and blanks. |
| C09: local implementation | The study team confirmed that C09 was introduced locally in 2022 and that the local response options included code 5 from its introduction. This local implementation differed from the option list in the supplied 2022 questionnaire. | The original recorded codes were retained. The local implementation statement was reported separately from the comparison of the two supplied questionnaire versions. |
| C09: availability and completion | C09 was not collected in the 2020–2021 development records. In an external city in Shandong Province, the item was available during 2022–2025, but completion was not mandatory in the local collection system. | Pre-2022 unavailability and blanks in an available item were distinguished in the missingness report. Both remained blank categorical inputs in the original model and were represented by the missing category. |
| C09: category absent from development | Code 5 was observed in 9 temporal-validation records and in no development or geographic-validation records. | The development-fitted encoder treated code 5 as an unseen category. Its corresponding C09 one-hot columns were all zero; the response was not converted into a blank or used to fit a new category effect. |
| D01; D03 | The available 2022 and 2025 questionnaires showed no identified wording or database coding difference. D03 asks about condom use at the most recent male anal intercourse during the past six months. D01 = 0 triggers a skip to F01 in both questionnaires. | D01 and D03 retained their binary response codes. D03 blanks were retained as a separate missing category, not changed to “no.” The observed blank pattern was compared with the D01-based skip instructions. |
| E01 | The available questionnaires specify a D01-based skip that passes over E01. In the external cohort, interviewers nevertheless asked E01 when D01 = 0 throughout all study years; recorded responses were actual interview responses. | Observed external E01 responses were retained. They were not overwritten on the basis of D01. External E01 blanks were not automatically classified as skip-related missingness. |
| F01; H01; I01; I03 | No wording or database coding difference was identified between the available 2022 and 2025 items. | The original binary response codes were retained and one-hot encoded. |
| G01 | The supplied 2022 questionnaire asks about current drug use, whereas the 2025 questionnaire asks whether drugs have ever been used. The study team confirmed that the local study question continued to ask about current drug use across study years and sources. | The variable was reported as current drug use. No conversion from current use to lifetime use was performed. |
| I02 | The available questionnaire items showed no identified wording or coding difference. The temporal-validation system initially displayed 0, but staff asked each participant and confirmed or changed the response. | Final recorded zeros were retained as “no,” rather than classified as unverified defaults or missing values. |
| All categorical predictors | Blank values and explicit unknown responses represent different recorded states. Refusal was not independently recorded. | Blank values were represented by a missing category. Explicit unknown codes were retained separately. Categories were learned from the relevant training data; unseen validation categories were ignored by the fitted one-hot encoder. |
| All numeric predictors | The original workflow treated numeric code 888 as missing. No numeric 888 values were observed for age or D02 in these cohorts. | Missing numeric values were replaced by the relevant training median, followed by standard scaling. Parameters were estimated without using the corresponding held-out records. |

**Part C. Feature sets used in sensitivity analyses**

| **Analysis** | **Feature count** | **C09 included** | **D02 included** | **D03 included** | **D04 included** |
| --- | --- | --- | --- | --- | --- |
| Primary model | 25 | Yes | Yes | Yes | No |
| Model without C09 | 24 | No | Yes | Yes | No |
| Model without D03 | 24 | Yes | Yes | No | No |
| Model without D02 and D03 | 23 | Yes | No | No | No |
| Model with D04 | 26 | Yes | Yes | Yes | Yes |

**Note:** Part A lists all 25 predictors used in the primary model. English item wording was translated from the available Chinese questionnaire documentation and supplemented by confirmed local implementation information. The codes shown are response codes, not scores of risk or knowledge. For binary items, the database codes are 1 and 0; these should not be confused with numbered response positions on a paper questionnaire. For C01–C08, code 1 means an affirmative response, not necessarily a correct answer.The comparison of questionnaire versions was based on the available 2022 and 2025 questionnaires. It does not establish that a complete copy of every source-year questionnaire was available. Confirmed local implementation details are stated separately in Part B. The reporting audit documented these differences without changing the original model inputs or preprocessing rules. Validation outcomes were not used to revise wording interpretations, predictor coding, or preprocessing.Predictors were restricted to information available before the current HIV test result was known. HIV screening and confirmatory results, post-test referrals, linkage records, follow-up variables, direct identifiers, and administrative record numbers were excluded. D04 was not a primary predictor and was evaluated only in the stated sensitivity analysis because of concern that it could act as a close proxy for the outcome. Raw missingness and predictor distributions are reported in Supplementary Table S4.

**Supplementary Table S4. **Raw predictor missingness, distributions, and preprocessing in the three cohorts****

****Part A. Raw blank counts and percentages for all primary predictors before preprocessing****

| **Predictor** | **Development, N = 1,585, n (%)** | **Temporal validation, N = 803, n (%)** | **Geographic external validation, N = 1,622, n (%)** |
| --- | --- | --- | --- |
| age | 0 (0.00) | 0 (0.00) | 0 (0.00) |
| D02 | 96 (6.06) | 67 (8.34) | 92 (5.67) |
| marital_status | 0 (0.00) | 0 (0.00) | 0 (0.00) |
| registered | 0 (0.00) | 0 (0.00) | 0 (0.00) |
| educational_level | 0 (0.00) | 0 (0.00) | 0 (0.00) |
| B07 | 0 (0.00) | 0 (0.00) | 0 (0.00) |
| B08 | 0 (0.00) | 0 (0.00) | 0 (0.00) |
| C01 | 0 (0.00) | 0 (0.00) | 0 (0.00) |
| C02 | 0 (0.00) | 0 (0.00) | 0 (0.00) |
| C03 | 0 (0.00) | 0 (0.00) | 0 (0.00) |
| C04 | 0 (0.00) | 0 (0.00) | 0 (0.00) |
| C05 | 0 (0.00) | 0 (0.00) | 0 (0.00) |
| C06 | 0 (0.00) | 0 (0.00) | 0 (0.00) |
| C07 | 0 (0.00) | 0 (0.00) | 0 (0.00) |
| C08 | 0 (0.00) | 0 (0.00) | 0 (0.00) |
| C09 | 784 (49.46) | 0 (0.00) | 504 (31.07) |
| D01 | 0 (0.00) | 0 (0.00) | 0 (0.00) |
| D03 | 96 (6.06) | 67 (8.34) | 92 (5.67) |
| E01 | 96 (6.06) | 67 (8.34) | 85 (5.24) |
| F01 | 0 (0.00) | 0 (0.00) | 0 (0.00) |
| G01 | 0 (0.00) | 0 (0.00) | 0 (0.00) |
| H01 | 0 (0.00) | 0 (0.00) | 0 (0.00) |
| I01 | 0 (0.00) | 0 (0.00) | 0 (0.00) |
| I02 | 0 (0.00) | 0 (0.00) | 0 (0.00) |
| I03 | 0 (0.00) | 0 (0.00) | 0 (0.00) |

**Part A reports blank source values before imputation, recoding, or one-hot encoding. It includes blanks caused by item unavailability or skip patterns. Explicit “do not know” responses for C01–C08 and “unsure” responses for C09 are not included in these blank counts; their counts and percentages are reported separately in Part C. The interpretation of blanks is described in Part D.**

****Part B. Observed distributions of numeric predictors before imputation****

| ****Predictor**** | ****Summary**** | ****Development, N = 1,585**** | ****Temporal validation, N = 803**** | ****Geographic external validation, N = 1,622**** |
| --- | --- | --- | --- | --- |
| **Age, years** | **Observed values, n** | **1,585** | **803** | **1,622** |
| **Age, years** | **Mean (SD)** | **31.46 (9.02)** | **29.65 (8.93)** | **31.62 (8.80)** |
| **Age, years** | **Median (Q1–Q3)** | **30 (25–36)** | **29 (23–34)** | **30 (25–37)** |
| **Age, years** | **Minimum–maximum** | **15–71** | **16–66** | **16–66** |
| **D02, male anal intercourse acts in the past week** | **Observed values, n** | **1,489** | **736** | **1,530** |
| **D02, male anal intercourse acts in the past week** | **Mean (SD)** | **1.78 (2.32)** | **1.84 (1.54)** | **1.94 (2.20)** |
| **D02, male anal intercourse acts in the past week** | **Median (Q1–Q3)** | **1 (1–2)** | **2 (1–2)** | **1 (1–2)** |
| **D02, male anal intercourse acts in the past week** | **Minimum–maximum** | **0–20** | **0–35** | **0–16** |

**Numeric summaries use observed values only. Blank values were excluded from these summaries and are reported in Part A. No numeric code 888, negative value, or noninteger value was observed for age or D02. SD, standard deviation; Q1 and Q3, first and third quartiles.**

**Part C. Raw categorical predictor distributions before recoding or one-hot encoding**

| ****Predictor**** | ****Response category or code**** | ****Development, N = 1,585, n (%)**** | ****Temporal validation, N = 803, n (%)**** | ****Geographic external validation, N = 1,622, n (%)**** |
| --- | --- | --- | --- | --- |
| **marital_status** | **1 = Unmarried** | **1,151 (72.62)** | **738 (91.91)** | **1,239 (76.39)** |
| **marital_status** | **2 = Married** | **340 (21.45)** | **32 (3.99)** | **281 (17.32)** |
| **marital_status** | **3 = Cohabiting** | **18 (1.14)** | **4 (0.50)** | **21 (1.29)** |
| **marital_status** | **4 = Divorced or widowed** | **76 (4.79)** | **29 (3.61)** | **81 (4.99)** |
| **registered** | **1 = Same province** | **1,404 (88.58)** | **765 (95.27)** | **1,456 (89.77)** |
| **registered** | **2 = Another province** | **179 (11.29)** | **37 (4.61)** | **162 (9.99)** |
| **registered** | **3 = Foreign nationality** | **2 (0.13)** | **1 (0.12)** | **4 (0.25)** |
| **educational_level** | **1 = Illiterate** | **4 (0.25)** | **7 (0.87)** | **2 (0.12)** |
| **educational_level** | **2 = Primary school** | **16 (1.01)** | **0 (0.00)** | **8 (0.49)** |
| **educational_level** | **3 = Junior middle school** | **139 (8.77)** | **33 (4.11)** | **105 (6.47)** |
| **educational_level** | **4 = Senior middle school or technical secondary school** | **484 (30.54)** | **607 (75.59)** | **558 (34.40)** |
| **educational_level** | **5 = Junior college or above** | **942 (59.43)** | **156 (19.43)** | **949 (58.51)** |
| **B07** | **1 = Homosexual** | **1,473 (92.93)** | **738 (91.91)** | **1,497 (92.29)** |
| **B07** | **2 = Heterosexual** | **9 (0.57)** | **5 (0.62)** | **14 (0.86)** |
| **B07** | **3 = Bisexual** | **91 (5.74)** | **47 (5.85)** | **102 (6.29)** |
| **B07** | **4 = Undetermined** | **12 (0.76)** | **13 (1.62)** | **9 (0.55)** |
| **B08** | **1 = Bar or dance hall** | **118 (7.44)** | **63 (7.85)** | **129 (7.95)** |
| **B08** | **2 = Tea house or club** | **14 (0.88)** | **2 (0.25)** | **16 (0.99)** |
| **B08** | **3 = Bathhouse** | **12 (0.76)** | **4 (0.50)** | **14 (0.86)** |
| **B08** | **4 = Park, public toilet, or outdoor grassy area** | **9 (0.57)** | **2 (0.25)** | **8 (0.49)** |
| **B08** | **5 = Internet or dating application** | **1,412 (89.09)** | **724 (90.16)** | **1,436 (88.53)** |
| **B08** | **6 = Other** | **20 (1.26)** | **8 (1.00)** | **19 (1.17)** |
| **C01** | **1 = Yes** | **1,557 (98.23)** | **793 (98.75)** | **1,598 (98.52)** |
| **C01** | **0 = No** | **9 (0.57)** | **3 (0.37)** | **7 (0.43)** |
| **C01** | **888 = Do not know** | **19 (1.20)** | **7 (0.87)** | **17 (1.05)** |
| **C02** | **1 = Yes** | **1,542 (97.29)** | **783 (97.51)** | **1,587 (97.84)** |
| **C02** | **0 = No** | **19 (1.20)** | **11 (1.37)** | **19 (1.17)** |
| **C02** | **888 = Do not know** | **24 (1.51)** | **9 (1.12)** | **16 (0.99)** |
| **C03** | **1 = Yes** | **205 (12.93)** | **25 (3.11)** | **210 (12.95)** |
| **C03** | **0 = No** | **1,323 (83.47)** | **750 (93.40)** | **1,362 (83.97)** |
| **C03** | **888 = Do not know** | **57 (3.60)** | **28 (3.49)** | **50 (3.08)** |
| **C04** | **1 = Yes** | **1,501 (94.70)** | **763 (95.02)** | **1,545 (95.25)** |
| **C04** | **0 = No** | **22 (1.39)** | **6 (0.75)** | **18 (1.11)** |
| **C04** | **888 = Do not know** | **62 (3.91)** | **34 (4.23)** | **59 (3.64)** |
| **C05** | **1 = Yes** | **1,537 (96.97)** | **773 (96.26)** | **1,576 (97.16)** |
| **C05** | **0 = No** | **11 (0.69)** | **16 (1.99)** | **13 (0.80)** |
| **C05** | **888 = Do not know** | **37 (2.33)** | **14 (1.74)** | **33 (2.03)** |
| **C06** | **1 = Yes** | **1,500 (94.64)** | **775 (96.51)** | **1,526 (94.08)** |
| **C06** | **0 = No** | **11 (0.69)** | **5 (0.62)** | **11 (0.68)** |
| **C06** | **888 = Do not know** | **74 (4.67)** | **23 (2.86)** | **85 (5.24)** |
| **C07** | **1 = Yes** | **1,540 (97.16)** | **770 (95.89)** | **1,562 (96.30)** |
| **C07** | **0 = No** | **6 (0.38)** | **6 (0.75)** | **7 (0.43)** |
| **C07** | **888 = Do not know** | **39 (2.46)** | **27 (3.36)** | **53 (3.27)** |
| **C08** | **1 = Yes** | **1,536 (96.91)** | **766 (95.39)** | **1,562 (96.30)** |
| **C08** | **0 = No** | **8 (0.50)** | **26 (3.24)** | **10 (0.62)** |
| **C08** | **888 = Do not know** | **41 (2.59)** | **11 (1.37)** | **50 (3.08)** |
| **C09** | **1 = None** | **168 (10.60)** | **14 (1.74)** | **165 (10.17)** |
| **C09** | **2 = Low** | **447 (28.20)** | **320 (39.85)** | **707 (43.59)** |
| **C09** | **3 = Average or moderate** | **148 (9.34)** | **411 (51.18)** | **203 (12.52)** |
| **C09** | **4 = High or very high** | **38 (2.40)** | **49 (6.10)** | **43 (2.65)** |
| **C09** | **5 = Unsure** | **0 (0.00)** | **9 (1.12)** | **0 (0.00)** |
| **C09** | **Blank** | **784 (49.46)** | **0 (0.00)** | **504 (31.07)** |
| **D01** | **1 = Yes** | **1,489 (93.94)** | **736 (91.66)** | **1,530 (94.33)** |
| **D01** | **0 = No** | **96 (6.06)** | **67 (8.34)** | **92 (5.67)** |
| **D03** | **1 = Yes** | **1,442 (90.98)** | **714 (88.92)** | **1,480 (91.25)** |
| **D03** | **0 = No** | **47 (2.97)** | **22 (2.74)** | **50 (3.08)** |
| **D03** | **Blank** | **96 (6.06)** | **67 (8.34)** | **92 (5.67)** |
| **E01** | **1 = Yes** | **99 (6.25)** | **88 (10.96)** | **129 (7.95)** |
| **E01** | **0 = No** | **1,390 (87.70)** | **648 (80.70)** | **1,408 (86.81)** |
| **E01** | **Blank** | **96 (6.06)** | **67 (8.34)** | **85 (5.24)** |
| **F01** | **1 = Yes** | **136 (8.58)** | **69 (8.59)** | **148 (9.12)** |
| **F01** | **0 = No** | **1,449 (91.42)** | **734 (91.41)** | **1,474 (90.88)** |
| **G01** | **1 = Yes** | **15 (0.95)** | **1 (0.12)** | **9 (0.55)** |
| **G01** | **0 = No** | **1,570 (99.05)** | **802 (99.88)** | **1,613 (99.45)** |
| **H01** | **1 = Yes** | **24 (1.51)** | **3 (0.37)** | **16 (0.99)** |
| **H01** | **0 = No** | **1,561 (98.49)** | **800 (99.63)** | **1,606 (99.01)** |
| **I01** | **1 = Yes** | **1,502 (94.76)** | **776 (96.64)** | **1,553 (95.75)** |
| **I01** | **0 = No** | **83 (5.24)** | **27 (3.36)** | **69 (4.25)** |
| **I02** | **1 = Yes** | **129 (8.14)** | **0 (0.00)** | **109 (6.72)** |
| **I02** | **0 = No** | **1,456 (91.86)** | **803 (100.00)** | **1,513 (93.28)** |
| **I03** | **1 = Yes** | **929 (58.61)** | **451 (56.16)** | **1,203 (74.17)** |
| **I03** | **0 = No** | **656 (41.39)** | **352 (43.84)** | **419 (25.83)** |

**All percentages use the full cohort denominator, including records with blank values. Categories with zero observations are shown when needed for comparison across cohorts. Blank rows are shown only for categorical predictors with observed blanks; Part A reports blank counts for every predictor. The blank counts repeated here are the same records reported in Part A and should not be added to them. Percentages may not total exactly 100 because of rounding. Full item wording and coding are provided in Supplementary Table S3.**

****Part D. Item availability, interpretation of blanks, and limits of missing-reason identification****

| ****Predictor or issue**** | ****Development cohort**** | ****Temporal-validation cohort**** | ****Geographic external-validation cohort**** | ****Interpretation**** |
| --- | --- | --- | --- | --- |
| **C09: item unavailable before introduction** | **784 (49.46%) records from 2020–2021** | **0 (0.00%)** | **0 (0.00%)** | **C09 was introduced in 2022. The 784 development blanks occurred before its introduction and are included in the total blank count.** |
| **C09: blank despite item availability** | **0 (0.00%)** | **0 (0.00%)** | **504 (31.07%)** | **The external collection system did not require completion of C09. These records had no recorded response to an available item. Individual reasons for leaving the field blank were not separately recorded.** |
| **C09: explicit unsure response** | **0 (0.00%)** | **9 (1.12%)** | **0 (0.00%)** | **Code 5 is an observed response category, not a blank. It was absent from development and handled as an unseen category in temporal validation.** |
| **D02: blanks consistent with the D01-based skip** | **96 (6.06%)** | **67 (8.34%)** | **92 (5.67%)** | **All observed D02 blanks occurred with D01 = 0, consistent with the available questionnaire skip instructions. Individual missing-reason flags were not separately recorded.** |
| **D03: blanks consistent with the D01-based skip** | **96 (6.06%)** | **67 (8.34%)** | **92 (5.67%)** | **All observed D03 blanks occurred with D01 = 0, consistent with the available questionnaire skip instructions. Blanks were not recoded as “no.”** |
| **E01: internal blanks consistent with the D01-based skip** | **96 (6.06%)** | **67 (8.34%)** | **Not applied** | **The internal blank pattern was consistent with the D01-based skip. The same classification was not imposed on the external cohort because its actual interview procedure differed.** |
| **E01: external blank responses** | **Not applicable** | **Not applicable** | **85 (5.24%)** | **Of these, 80 were confirmed as unanswered. The specific reason for the remaining 5 was not separately confirmed. None was automatically classified as a skip solely because D01 = 0.** |
| **E01: external responses when D01 = 0** | **Not applicable** | **Not applicable** | **87 recorded responses** | **Interviewers asked E01 even when D01 = 0 throughout 2022–2025. These were actual interview responses and were retained in the model inputs.** |
| **C01–C08: explicit unknown responses** | **Reported separately for each item in Part C** | **Reported separately for each item in Part C** | **Reported separately for each item in Part C** | **Code 888 means “do not know.” These responses were kept separate from blank values.** |
| **B07: undetermined response** | **12 (0.76%)** | **13 (1.62%)** | **9 (0.55%)** | **Code 4 is a substantive questionnaire response option. It was retained as a category and was not counted as a blank.** |
| **I02: recorded no responses** | **1,456 (91.86%)** | **803 (100.00%)** | **1,513 (93.28%)** | **In temporal validation, staff confirmed or changed the initial displayed value after asking each participant. Final zeros were retained as actual “no” responses.** |
| **Refused responses** | **Not separately identifiable** | **Not separately identifiable** | **Not separately identifiable** | **Refusal was not recorded as an independent response code or reason. A total refusal count cannot be inferred from the blank counts.** |

**The rows in Part D explain the counts in Parts A and C; they are not additional missing records. C09 blanks are separated into pre-introduction unavailability and blanks in an available item. For D02, D03, and internal E01, consistency with the skip instructions describes the observed data pattern and does not imply that a separate reason was recorded for every blank. External E01 was handled according to the confirmed local interview procedure. No assumption of random missingness was made.**

****Part E. Preprocessing and handling of predictor values****

| **Predictor or data state** | **Handling in the original modeling workflow** |
| --- | --- |
| Age and D02 | Numeric conversion, median imputation, and standard scaling. The median and scaling parameters were estimated from the relevant training records. |
| Numeric code 888 | Converted to missing by the original numeric preprocessing rule. No such values were observed for age or D02 in the three cohorts. |
| Recorded zero for D02 | Retained as a numeric value; not automatically changed to missing. |
| Blank categorical values | Represented by an explicit missing category before one-hot encoding. |
| C01–C08 code 888 | Retained as a separate categorical unknown response, distinct from the missing category. |
| C09 code 5 | Retained as an explicit “unsure” response. In temporal validation, it was handled by the fitted encoder as an unseen category because it was absent from development. |
| Categorical encoding | Categories were learned from the relevant training records and one-hot encoded. |
| Unseen validation categories | Encoded as zeros across the corresponding predictor's one-hot columns. Validation data were not used to add or fit categories. |
| D03 blanks | Retained as categorical missing values; not converted into “no.” |
| External E01 responses | Observed responses were retained, including those recorded when D01 = 0. |
| Temporal-validation I02 zeros | Retained as “no,” consistent with the confirmed interview procedure. |
| Cross-fitting | Each held-out set was excluded from fitting the preprocessing used for its predictions. |
| Final validation | The development-fitted preprocessing was applied unchanged to each validation cohort. |
| Reporting audit | Raw missingness and distributions were summarized without changing the original analytic inputs or refitting the prediction models. |

**Note:** The development, temporal-validation, and geographic external-validation cohorts included 1,585, 803, and 1,622 participants, respectively, with one analyzed record per participant. Missingness and predictor distributions were summarized from the source values before imputation, recoding, or one-hot encoding.Part A reports raw blanks for all 25 primary predictors. Part B summarizes observed numeric values. Part C reports categorical response distributions, including explicit unknown responses and observed blanks. Part D distinguishes documented item unavailability from blanks in an available item and describes skip-related patterns and limits of reason identification. Part E reports the original preprocessing rules. Blank entries, explicit unknown responses, and refusals were not treated as interchangeable states. Refusal could not be counted separately because it was not independently recorded.Percentages in Parts A, C, and the cohort-specific count entries in Part D use the full cohort denominator unless otherwise stated. Numeric summaries use the observed values only. Counts repeated across parts describe the same records and must not be added together. The reporting audit did not change the locked model, calibration, or threshold. Validation outcomes were not used to redefine predictor categories or missing-data handling.

**Supplementary Table S5. Model development, selection, calibration, and locking procedure.**

## Part A. Candidate architectures

| **Architecture** | **Analytical role** | **Development procedure** | **Included in locked primary stack** |
| --- | --- | --- | --- |
| Penalized logistic regression | Conventional linear model and base learner | Stratified cross-fitting within development data | Yes |
| Random forest | Non-linear tree-based base learner | Stratified cross-fitting within development data | Yes |
| HistGradientBoosting | Gradient-boosting candidate and base learner | Stratified cross-fitting within development data | Yes |
| Stacked ensemble | Candidate model selected for the primary locked workflow | Logistic-regression meta-learner trained on nested out-of-fold base-model predictions | Selected primary model |
| TabNet | Exploratory tabular deep-learning candidate | Evaluated during exploratory model development | No |

## Part B. Models included in the final development-stage comparison

| **Architecture** | **Evaluation source** | **Calibrated AUROC** | **Calibrated AUPRC** | **Calibrated Brier score** | **Brier skill score** | **Selected** |
| --- | --- | --- | --- | --- | --- | --- |
| HistGradientBoosting | Calibrated outer cross-fitted development predictions | 0.841 | 0.368 | 0.0357 | 0.187 | No |
| Stacked ensemble | Calibrated outer cross-fitted development predictions | 0.855 | 0.416 | 0.0331 | 0.247 | Yes |

## Part C. Model-locking rules

| **Item** | **Locked specification** |
| --- | --- |
| Development period | 2020–2023 |
| Development cohort size | 1,585 encounters |
| Development confirmed events | 73 |
| Outer-fold strategy | Stratified five-fold cross-fitting |
| Stacking strategy | Nested out-of-fold prediction |
| Meta-learner | Logistic regression |
| Probability calibration | Cross-fitted Platt calibration using development data |
| Reported performance measures | AUROC, AUPRC, Brier score, and Brier skill score |
| Model-selection rule | Retain candidates within 0.01 of the highest calibrated development AUROC. Among these candidates, retain those within 0.001 of the lowest Brier score. Apply the stated preference order if more than one candidate remains. |
| Primary comparison preference | HistGradientBoosting before the stacked ensemble |
| Expanded comparison preference | Penalized logistic regression, HistGradientBoosting, random forest, then the stacked ensemble |
| Role of AUPRC and Brier skill score | Reported performance measures; not additional selection steps |
| Operating-threshold derivation | 90th percentile of calibrated outer cross-fitted development scores, using linear interpolation |
| Exact locked threshold | 0.09438076500258265 |
| Threshold operator | Score greater than or equal to the exact locked threshold |
| Random seed | Master seed 42; deterministic fold-specific seeds |
| Refitting in temporal validation | No |
| Refitting in geographic external validation | No |
| Recalibration in temporal validation | No |
| Recalibration in geographic external validation | No |
| Threshold re-estimation in validation | No |

## Part D. Nested fitting structure and model settings

| Component | Setting or procedure |
| --- | --- |
| Outer evaluation | Stratified five-fold cross-fitting with shuffling |
| Calibration training | Five-fold raw-score cross-fitting within each outer training set |
| Meta-learner training | Five-fold base-model out-of-fold predictions within each raw stack fit |
| Penalized logistic regression | L2 penalty; C = 1.0; liblinear solver; balanced class weights; maximum 5,000 iterations |
| Random forest | 500 trees; maximum depth 8; minimum leaf size 5; square-root feature sampling; balanced_subsample class weights |
| HistGradientBoosting | Learning rate 0.04; 250 iterations; maximum 15 leaf nodes; minimum leaf size 12; L2 regularization 0.1 |
| Logistic meta-learner | L2 penalty; C = 1.0; liblinear solver; no class weighting; maximum 5,000 iterations |
| Platt calibrator | Logistic regression on the raw-score logit; L2 penalty; C = 1,000,000; lbfgs solver; no class weighting; maximum 5,000 iterations |
| Hyperparameter search | None in the revised analysis; the original base-model settings were retained |
| Numeric preprocessing | Median imputation followed by standard scaling, fitted using the relevant training records |
| Categorical preprocessing | Explicit missing categories and one-hot encoding; unseen categories ignored by the fitted encoder |
| Final development fit | Internal cross-fitting retained for meta-learning and calibration; base learners refitted on all 1,585 development records |
| Randomization | Master seed 42; deterministic fold-specific seeds; matching comparisons used shared splits |

**Part E. Absolute performance of calibrated candidate models**

| **Cohort** | **Model** | **AUROC** | **AUPRC** | **Brier score** | **Brier skill score** | **Calibration-in-the-large (95% CI)** | **Calibration slope (95% CI)** |
| --- | --- | --- | --- | --- | --- | --- | --- |
| **Development** | **LR** | **0.826** | **0.390** | **0.0343** | **0.219** | **−0.036 (−0.159 to 0.086)** | **0.941 (0.737–1.194)** |
| **Development** | **HGB** | **0.841** | **0.368** | **0.0357** | **0.187** | **−0.131 (−0.236 to −0.027)** | **1.025 (0.828–1.239)** |
| **Development** | **RF** | **0.879** | **0.452** | **0.0325** | **0.260** | **−0.061 (−0.191 to 0.060)** | **0.993 (0.805–1.212)** |
| **Development** | **Stack** | **0.855** | **0.416** | **0.0331** | **0.247** | **−0.022 (−0.175 to 0.121)** | **0.933 (0.783–1.102)** |
| **Temporal validation** | **LR** | **0.948** | **0.622** | **0.0189** | **0.456** | **−0.570 (−0.862 to −0.325)** | **1.296 (0.973–1.842)** |
| **Temporal validation** | **HGB** | **0.938** | **0.626** | **0.0203** | **0.418** | **−0.328 (−0.512 to −0.164)** | **1.551 (1.153–2.213)** |
| **Temporal validation** | **RF** | **0.964** | **0.718** | **0.0172** | **0.505** | **−0.014 (−0.243 to 0.199)** | **1.506 (1.143–2.096)** |
| **Temporal validation** | **Stack** | **0.963** | **0.696** | **0.0165** | **0.527** | **−0.118 (−0.338 to 0.089)** | **1.530 (1.206–2.064)** |
| **Geographic external validation** | **LR** | **0.953** | **0.656** | **0.0171** | **0.451** | **−0.381 (−0.538 to −0.229)** | **1.440 (1.175–1.824)** |
| **Geographic external validation** | **HGB** | **0.960** | **0.733** | **0.0163** | **0.473** | **−0.519 (−0.639 to −0.408)** | **1.765 (1.504–2.188)** |
| **Geographic external validation** | **RF** | **0.977** | **0.694** | **0.0168** | **0.459** | **−0.333 (−0.498 to −0.186)** | **1.435 (1.236–1.724)** |
| **Geographic external validation** | **Stack** | **0.974** | **0.746** | **0.0153** | **0.507** | **−0.357 (−0.523 to −0.205)** | **1.414 (1.245–1.663)** |

**Part E note: All models used calibrated scores. Development estimates used outer cross-fitted predictions. Validation estimates used models fitted on the development cohort and applied without refitting or recalibration. Calibration confidence intervals are percentile intervals from 1,000 stratified encounter-level bootstrap samples of fixed predictions. They do not include uncertainty from refitting the workflow. Calibration-in-the-large and calibration slope have ideal values of 0 and 1, respectively. Supplementary Table S18 reports paired differences and their confidence intervals.**

**LR, penalized logistic regression; HGB, HistGradientBoosting; RF, random forest; Stack, stacked ensemble.**

**Note:** The stacked ensemble was selected in the primary two-model comparison using the stated development-only rule. The expanded four-model comparison selected random forest and was reported as an additional analysis. It did not replace the primary model. The revised analysis did not establish that the selection rule had been prospectively specified before the original results were examined. This selection was based on development data only. OOF denotes out-of-fold. Matching candidate and sensitivity comparisons used the same development records and shared fold assignments where required. TabNet was evaluated as an exploratory candidate but was not included in the locked primary stack. Neither validation cohort was used to fit a base learner, meta-learner, probability calibrator, or operating threshold.

**Supplementary Table S6. Temporal and geographic validation performance of the locked calibrated stacked ensemble.**

| **Measure** | **Temporal validation, 2024–2025** | **Geographic external validation, 2022–2025** |
| --- | --- | --- |
| Testing encounters, n | 803 | 1,622 |
| Confirmed events, n | 29 | 52 |
| Event prevalence, % | 3.6 | 3.2 |
| AUROC | 0.963 | 0.974 |
| AUPRC | 0.696 | 0.746 |
| Brier score | 0.0165 | 0.0153 |
| Brier skill score | 0.527 | 0.507 |
| Calibration-in-the-large | −0.118 | −0.357 |
| Joint calibration intercept | 1.107 | 0.408 |
| Calibration slope | 1.530 | 1.414 |
| Development-locked score threshold | 0.0944 | 0.0944 |
| Encounters selected, n | 45 | 103 |
| Encounters selected, % | 5.6 | 6.4 |
| Sensitivity, % | 75.9 | 86.5 |
| Specificity, % | 97.0 | 96.3 |
| Positive predictive value, % | 48.9 | 43.7 |
| Negative predictive value, % | 99.1 | 99.5 |
| True positives, n | 22 | 45 |
| False positives, n | 23 | 58 |
| False negatives, n | 7 | 7 |
| True negatives, n | 751 | 1,512 |

**Note: The locked calibrated stacked ensemble was applied without refitting or recalibration. The exact threshold was 0.09438076500258265 and was applied unchanged in both cohorts. AUPRC was summarized using average precision. A positive Brier skill score indicates lower prediction error than a constant prediction equal to the event prevalence in the relevant cohort. Calibration-in-the-large was estimated with the score-logit coefficient fixed at 1. The joint intercept and slope were estimated together in a separate logistic model. These assessments did not update predictions. The observed calibration deviations support using the scores for relative prioritization rather than as exact individual probabilities. Confidence intervals are reported in Supplementary Tables S8 and S9.**

**Supplementary Table S7. Cross-fitted development performance of the selected model.**

| **Cohort and evaluation method** | **Testing encounters, n** | **Confirmed HIV-positive events, n** | **Event prevalence, %** | **AUROC** | **AUPRC** | **Brier score** | **Brier skill score** | **Calibration-in-the-large** | **Joint calibration intercept** | **Calibration slope** |
| --- | --- | --- | --- | --- | --- | --- | --- | --- | --- | --- |
| Nantong development cohort, calibrated outer cross-fitted evaluation | 1,585 | 73 | 4.6 | 0.855 | 0.416 | 0.0331 | 0.247 | −0.022 | −0.170 | 0.933 |

**Note: Development estimates used calibrated outer cross-fitted predictions for the selected stacked architecture. Each held-out encounter was excluded from fitting its preprocessing, base learners, meta-learner, and calibrator. These estimates are not apparent in-sample performance. The architecture was selected using the combined development predictions, so this table does not by itself provide a fully selection-adjusted estimate of the selection procedure. Table 2 reports confidence intervals. Supplementary Table S6 reports the separate temporal and geographic validation results. Calibration-in-the-large and the joint calibration intercept are distinct estimates. OOF, out-of-fold; AUROC, area under the receiver operating characteristic curve; AUPRC, area under the precision–recall curve.**

**Supplementary Table S8. Bootstrap confidence intervals and calibration estimates.**

| **Cohort** | **Measure** | **Estimate** | **95% CI** | **Bootstrap repetitions** |
| --- | --- | --- | --- | --- |
| Temporal validation | AUROC | 0.963 | 0.930–0.990 | 1,000 |
| Temporal validation | AUPRC | 0.696 | 0.536–0.880 | 1,000 |
| Temporal validation | Brier score | 0.0165 | 0.0119–0.0215 | 1,000 |
| Temporal validation | Brier skill score | 0.527 | 0.382–0.659 | 1,000 |
| Temporal validation | Calibration-in-the-large | −0.118 | −0.338 to 0.089 | 1,000 |
| Temporal validation | Joint calibration intercept | 1.107 | 0.197–2.393 | 1,000 |
| Temporal validation | Calibration slope | 1.530 | 1.206–2.064 | 1,000 |
| Geographic external validation | AUROC | 0.974 | 0.955–0.989 | 1,000 |
| Geographic external validation | AUPRC | 0.746 | 0.652–0.842 | 1,000 |
| Geographic external validation | Brier score | 0.0153 | 0.0120–0.0186 | 1,000 |
| Geographic external validation | Brier skill score | 0.507 | 0.402–0.613 | 1,000 |
| Geographic external validation | Calibration-in-the-large | −0.357 | −0.523 to −0.205 | 1,000 |
| Geographic external validation | Joint calibration intercept | 0.408 | 0.011–0.969 | 1,000 |
| Geographic external validation | Calibration slope | 1.414 | 1.245–1.663 | 1,000 |

**Note: Percentile 95% confidence intervals were calculated from 1,000 stratified encounter-level bootstrap samples of fixed predictions. Calibration-in-the-large fixes the coefficient of the score logit at 1. The joint calibration intercept and slope are estimated together in a separate logistic model. The joint intercept is not calibration-in-the-large. These models assessed calibration without updating predictions. The intervals do not include uncertainty from refitting the full modeling workflow.**

**Supplementary Table S9. Performance at the development-locked score threshold of 0.0944.**

| **Measure** | **Temporal validation** | **Geographic external validation** |
| --- | --- | --- |
| Total encounters, n | 803 | 1,622 |
| Confirmed events, n | 29 | 52 |
| Non-events, n | 774 | 1,570 |
| Encounters selected, n | 45 | 103 |
| Encounters selected, % | 5.6 (4.4–7.0) | 6.4 (5.4–7.3) |
| True positives, n | 22 | 45 |
| False positives, n | 23 | 58 |
| False negatives, n | 7 | 7 |
| True negatives, n | 751 | 1,512 |
| Sensitivity, % | 75.9 (58.6–89.7) | 86.5 (76.9–96.2) |
| Specificity, % | 97.0 (95.7–98.2) | 96.3 (95.3–97.3) |
| Positive predictive value, % | 48.9 (38.2–61.5) | 43.7 (37.4–51.5) |
| Negative predictive value, % | 99.1 (98.4–99.6) | 99.5 (99.2–99.9) |

**Note: The threshold was the 90th percentile of calibrated outer cross-fitted development scores, using linear interpolation. The exact locked value was 0.09438076500258265. Scores at or above this value were selected. Values in parentheses are percentile 95% confidence intervals from 1,000 stratified encounter-level bootstrap samples of fixed predictions. The locked threshold was applied unchanged in both validation cohorts. No validation outcome was used to revise the model, calibration, or threshold. A positive threshold result indicates that the encounter was prioritized for possible additional support; it does not indicate a diagnostic HIV result or restrict access to testing and standard care.**

**Supplementary Table S10. Fixed-workload ranking analyses for optional additional support.**

| **Validation cohort** | **Nominal workload** | **Prioritized encounters, n** | **Confirmed events among prioritized encounters, n/N (%)** | **Observed event proportion among prioritized encounters, %** | **Encounters per captured event** |
| --- | --- | --- | --- | --- | --- |
| Temporal validation | 5% | 41 | 22/29 (75.9) | 53.7 | 1.86 |
| Temporal validation | 10% | 81 | 25/29 (86.2) | 30.9 | 3.24 |
| Temporal validation | 20% | 161 | 26/29 (89.7) | 16.1 | 6.19 |
| Temporal validation | 30% | 241 | 28/29 (96.6) | 11.6 | 8.61 |
| Geographic external validation | 5% | 82 | 41/52 (78.8) | 50.0 | 2.00 |
| Geographic external validation | 10% | 163 | 46/52 (88.5) | 28.2 | 3.54 |
| Geographic external validation | 20% | 325 | 50/52 (96.2) | 15.4 | 6.50 |
| Geographic external validation | 30% | 487 | 51/52 (98.1) | 10.5 | 9.55 |

**Note: Encounters were ranked from the highest to the lowest locked-model score. At each nominal workload, the number prioritized was calculated by multiplying the cohort size by the workload proportion and rounding up to the next whole encounter. Score ties were resolved using the original record order. Fixed-workload analyses were descriptive and did not contribute to model fitting, model selection, probability calibration, or threshold selection. They describe possible prioritization for optional additional support and do not restrict HIV testing, laboratory confirmation, routine counseling, prevention services, or clinical care.**

**Supplementary Table S11. Rationale for the handling of decision-curve analysis.**

| **Item** | **Description** |
| --- | --- |
| Intended use | Prioritization of encounters for possible additional counseling, prevention assessment, peer support, or follow-up |
| Diagnostic use | Not intended |
| Treatment-allocation use | Not intended |
| Access restriction | The model must not be used to restrict or delay HIV testing or other standard services |
| Nature of decision | Allocation of optional additional support under limited service capacity |
| Primary operational evidence | Performance at the locked threshold and event capture under fixed workload levels |
| Decision-curve role | Formal decision-curve analysis was not used to support a claim of clinical net benefit |
| Formal net-benefit claim | None |
| Reason for caution | The score did not represent a treatment threshold, and the relative benefits and harms of additional support were not prospectively quantified |
| Required next step | Prospective workflow evaluation before routine service use |

**Note: The model was not designed to replace HIV testing, laboratory confirmation, professional judgment, or standard counseling.** Because the consequences and relative value of providing additional support were not prospectively quantified, no formal claim of improved clinical net benefit was made.

**Supplementary Table S12. Distribution differences and annual descriptive performance.**

**Part A. Summary of distribution differences**

| **Audit domain** | **Comparison** | **Main observation** | **Interpretation** |
| --- | --- | --- | --- |
| Questionnaire version | Across years and study settings | Questionnaire wording, response options, and local implementation differences are described in Supplementary Table S3. | Possible instrument-related differences were documented without changing the original model inputs. |
| Educational level | Development versus later records | Category frequencies differed; the study team confirmed consistent meanings for codes 4 and 5 across years and sources. | The original codes were retained. The descriptive distributions do not establish the reasons for these differences. |
| Marital status | Development versus later records | Distribution changes identified | Requires monitoring rather than causal interpretation |
| C09 | Across years and study settings | The item was unavailable in 2020–2021. It was not mandatory in the external collection system during 2022–2025. Code 5 occurred in temporal validation but not in development. | Original categorical handling was retained. Raw blanks and response distributions are reported in Supplementary Table S4; removal results are reported in Supplementary Table S14. |
| D02 | Across years and study settings | Distribution differences identified | May reflect coding or population differences |
| D03 | Across years and study settings | Distribution differences identified | Retained after protocol review; removal examined |
| D04 | Across years and study settings | Strong outcome-related pattern and proxy concern | Excluded from the primary model |
| I03 and related prevention items | Across years and study settings | Distribution changes identified | May reflect prevention uptake, questionnaire practice, or both |
| Missingness | Across periods and settings | Raw blank counts and percentages differed by predictor and cohort. Explicit unknown responses were reported separately. | Supplementary Table S4 distinguishes item unavailability, blanks, and unknown responses where supported; refusal could not be separately identified. |
| Predicted scores | Development versus validation cohorts | Score distributions differed | No validation-based threshold revision was performed |
| Outcome prevalence | Development versus temporal validation | 4.6% versus 3.6% | Lower prevalence in later Nantong records |
| Outcome prevalence | Development versus geographic external validation | 4.6% versus 3.2% | Lower prevalence in the external setting |
| Discrimination | Temporal and geographic validation | High in the separately analyzed temporal and geographic validation cohorts | Discrimination was high in both cohorts; service benefit was not assessed |
| Calibration | Temporal and geographic validation | Calibration-in-the-large indicated overall overprediction in geographic validation. Calibration slopes exceeded 1 in both validation cohorts. | Scores should not be treated as exact probabilities |

**Part B. Annual descriptive performance of the selected model**

| **Cohort** | **Year** | **Encounters, n** | **Events, n** | **AUROC** | **AUPRC** | **Brier score** | **Brier skill score** |
| --- | --- | --- | --- | --- | --- | --- | --- |
| **Development** | **2020** | **395** | **27** | **0.626** | **0.164** | **0.0652** | **−0.024** |
| **Development** | **2021** | **389** | **12** | **0.824** | **0.462** | **0.0214** | **0.286** |
| **Development** | **2022** | **392** | **16** | **0.826** | **0.327** | **0.0344** | **0.122** |
| **Development** | **2023** | **409** | **18** | **0.995** | **0.914** | **0.0120** | **0.714** |
| **Temporal validation** | **2024** | **409** | **17** | **0.988** | **0.861** | **0.0164** | **0.588** |
| **Temporal validation** | **2025** | **394** | **12** | **0.928** | **0.579** | **0.0165** | **0.440** |
| **Geographic external validation** | **2022** | **400** | **13** | **0.950** | **0.697** | **0.0180** | **0.429** |
| **Geographic external validation** | **2023** | **400** | **13** | **0.968** | **0.663** | **0.0179** | **0.429** |
| **Geographic external validation** | **2024** | **410** | **13** | **0.985** | **0.842** | **0.0112** | **0.636** |
| **Geographic external validation** | **2025** | **412** | **13** | **0.989** | **0.794** | **0.0142** | **0.534** |

**Note:** These findings summarise the distribution-shift audit and should not be interpreted as proof of a true epidemiological change. Differences may reflect questionnaire revisions, coding practice, prevention uptake, or changes in the population seeking testing. Future use should include regular monitoring of missingness, category frequencies, score distributions, event capture, and calibration. Annual development estimates used the corresponding subsets of calibrated outer cross-fitted predictions. Annual validation estimates used the locked model without refitting. No model was fitted separately for an individual year. These estimates are descriptive and are based on small annual event counts. They do not establish the cause of performance differences across years.

**Supplementary Table S13. Validation prediction scores and data governance.**

| **Item** | **Description** |
| --- | --- |
| Prediction source | Locked calibrated stacked ensemble |
| Cohorts represented | Nantong temporal validation and geographic external validation |
| Main audit fields | Cohort, year, binary outcome, locked-model score, and fields required for performance and audit calculations |
| Analytical use | Discrimination, calibration, threshold, confusion-matrix, and fixed-workload analyses |
| Model refitting from validation scores | No |
| Probability recalibration from validation scores | No |
| Threshold revision from validation scores | No |
| Direct identifiers | Excluded |
| Record-level rows reproduced in this supplement | No |
| Public availability | Record-level prediction files are not publicly available |
| Reason for restriction | sensitive health and behavioral information relating to a key population, together with institutional and inter-institutional data-governance requirements |
| Possible access route | A reasonable request for controlled access may be considered after approval by the relevant data-controlling institutions and confirmation that the proposed use complies with the applicable data-use agreement |

**Note:** Even after direct identifiers have been removed, record-level outcomes and behavioral data from a key population remain sensitive. The prediction-score files are therefore retained in the controlled institutional analysis archive and are not reproduced in this supplementary document.

**Supplementary Table S14. Sensitivity analyses.**

**Part A. Validation performance of the primary and altered-feature models**

| **Analysis** | **Validation cohort** | **N** | **Events** | **AUROC** | **AUPRC** | **Brier score** | **Brier skill score** | **Calibration-in-the-large** | **Joint calibration intercept** | **Calibration slope** |
| --- | --- | --- | --- | --- | --- | --- | --- | --- | --- | --- |
| Primary model | Temporal validation | 803 | 29 | 0.963 | 0.696 | 0.0165 | 0.527 | −0.118 | 1.107 | 1.530 |
| Primary model | Geographic external validation | 1,622 | 52 | 0.974 | 0.746 | 0.0153 | 0.507 | −0.357 | 0.408 | 1.414 |
| Without C09 | Temporal validation | 803 | 29 | 0.932 | 0.610 | 0.0200 | 0.427 | 0.147 | 0.887 | 1.270 |
| Without C09 | Geographic external validation | 1,622 | 52 | 0.949 | 0.687 | 0.0170 | 0.453 | −0.410 | 0.024 | 1.242 |
| Without D03 | Temporal validation | 803 | 29 | 0.890 | 0.472 | 0.0252 | 0.277 | −0.424 | 0.383 | 1.416 |
| Without D03 | Geographic external validation | 1,622 | 52 | 0.939 | 0.534 | 0.0219 | 0.296 | −0.278 | 1.163 | 1.687 |
| Without D02 and D03 | Temporal validation | 803 | 29 | 0.865 | 0.426 | 0.0267 | 0.232 | −0.442 | 0.525 | 1.471 |
| Without D02 and D03 | Geographic external validation | 1,622 | 52 | 0.927 | 0.497 | 0.0225 | 0.276 | −0.339 | 1.047 | 1.647 |
| With D04 | Temporal validation | 803 | 29 | 0.977 | 0.686 | 0.0174 | 0.499 | 0.388 | 0.564 | 1.070 |
| With D04 | Geographic external validation | 1,622 | 52 | 0.996 | 0.916 | 0.0098 | 0.685 | −0.405 | 0.004 | 1.955 |

**Part B. Paired AUROC differences after predictor removal**

| **Analysis** | **Cohort** | **AUROC difference versus the primary model** | **95% CI** |
| --- | --- | --- | --- |
| Without C09 | Temporal validation | −0.032 | −0.070 to −0.001 |
| Without C09 | Geographic external validation | −0.025 | −0.049 to −0.005 |
| Without D03 | Temporal validation | −0.073 | −0.129 to −0.026 |
| Without D03 | Geographic external validation | −0.035 | −0.064 to −0.010 |
| Without D02 and D03 | Temporal validation | −0.098 | −0.171 to −0.037 |
| Without D02 and D03 | Geographic external validation | −0.047 | −0.078 to −0.021 |

**Note: Sensitivity analyses retained the selected stacked architecture and changed only the stated predictor set. Each workflow was refitted and calibrated using development data only, with the same nested structure and shared fold assignments where paired comparison was required. Calibration-in-the-large was estimated with the score-logit coefficient fixed at 1. The joint intercept and slope were estimated together in a separate logistic model. These calibration assessments did not update predictions. The sensitivity models did not replace or update the locked primary model.Performance decreased after C09, D03, or both D02 and D03 were removed. These comparisons describe changes in predictive performance after refitting the workflow with the stated predictor sets and should not be interpreted as causal effects. Removing C09 or D03 removed all encoded categories of that predictor, including its missing category. Related information could remain in other predictors, including D01. These analyses did not separate the contribution of observed responses from that of missingness patterns. Item definitions and implementation differences are reported in Supplementary Table S3, and raw missingness and response distributions are reported in Supplementary Table S4.D04 was excluded from the primary model because its coding pattern raised concern that it could act as an outcome proxy. Adding D04 in the sensitivity analysis improved several performance measures, especially in geographic external validation. This improvement did not resolve the proxy concern and was not used to change the primary predictor set.Differences were calculated as the altered-feature model minus the primary model. Percentile 95% confidence intervals used 1,000 paired bootstrap samples, with encounters resampled within outcome groups. Both models used the same sampled encounters, and predictions remained fixed. The intervals do not include uncertainty from refitting the full workflow and were not adjusted for multiple comparisons. Negative AUROC differences indicate lower discrimination after predictor removal.Differences were calculated from unrounded estimates and may differ slightly from differences between the rounded values shown in Part A.**

**Supplementary Table S15. Numerical consistency, data separation, and model-locking checklist.**

| **Audit item** | **Expected or locked value** | **Observed value** | **Status** |
| --- | --- | --- | --- |
| Nantong source records | 2,460 | 2,460 | Matched |
| Previously known HIV-positive exclusions | 38 | 38 | Matched |
| Unresolved outcome or calendar-year exclusions | 34 | 34 | Matched |
| Nantong analytic records | 2,388 | 2,388 | Matched |
| Nantong confirmed events | 102 | 102 | Matched |
| Development records | 1,585 | 1,585 | Matched |
| Development events | 73 | 73 | Matched |
| Temporal validation records | 803 | 803 | Matched |
| Temporal validation events | 29 | 29 | Matched |
| Geographic external validation records | 1,622 | 1,622 | Matched |
| Geographic external validation events | 52 | 52 | Matched |
| Primary feature count | 25 | 25 | Matched |
| D04 excluded from primary model | Yes | Yes | Matched |
| Selected architecture | Stacked ensemble | Stacked ensemble | Matched |
| Locked threshold | 0.09438076500258265 | 0.09438076500258265 | Matched |
| Master random seed | 42 | 42 | Matched |
| Bootstrap repetitions | 1,000 | 1,000 | Matched |
| Shared outer-fold assignments used for paired comparisons where required | Yes | Yes | Matched |
| Shared calibration-fold assignments used for paired comparisons where required | Yes | Yes | Matched |
| Training and holdout record-index overlap in the logged fitting procedures | 0 | 0 | Matched |
| Paired candidate-model bootstrap | Yes | Yes | Matched |
| Paired sensitivity-analysis bootstrap | Yes | Yes | Matched |
| Temporal data used for model fitting | No | No | Matched |
| External data used for model fitting | No | No | Matched |
| Validation data used for calibration | No | No | Matched |
| Validation data used for model selection | No | No | Matched |
| Validation data used for threshold selection | No | No | Matched |
| Threshold re-estimated in validation | No | No | Matched |
| Temporal AUROC | 0.963 | 0.963 | Matched |
| Geographic external AUROC | 0.974 | 0.974 | Matched |
| Temporal AUPRC | 0.696 | 0.696 | Matched |
| Geographic external AUPRC | 0.746 | 0.746 | Matched |
| Temporal threshold true positives | 22 | 22 | Matched |
| Geographic external threshold true positives | 45 | 45 | Matched |
| Temporal top-10% event capture | 25/29 | 25/29 | Matched |
| Geographic external top-10% event capture | 46/52 | 46/52 | Matched |
| All logged fits exclude ancestor holdouts | Yes | Yes | Matched |
| Number of logged fits | 6,490 | 6,490 | Matched |
| Number of logged splits | 1,975 | 1,975 | Matched |
| Bootstrap diagnostic failure records | 0 | 0 | Matched |
| Validation results examined in the original analysis | Yes | Yes | Matched |

**Note: The fold audit found no overlap between training and holdout record indices in the logged fitting procedures. This was a record-index check, not an identity-linkage check. Disease control staff completed person-level deduplication upstream and retained the first recorded encounter for each participant, as described in Section 2.2 and Supplementary Table S1. Shared outer-fold and calibration-fold assignments were used where paired model or sensitivity comparisons required them. The fitting and selection checks refer to the revised analysis; the validation results had already been examined in the original analysis.**

**Supplementary Table S16. Software environment and reproducibility settings.**

| **Software, package, or setting** | **Version or value** |
| --- | --- |
| Analysis version | Reviewer-34 revised analysis |
| Python | 3.10.21 |
| Operating platform | Linux 7.0.0-31-generic, x86_64, glibc 2.39 |
| NumPy | 2.2.6 |
| pandas | 2.3.3 |
| scikit-learn | 1.7.2 |
| SciPy | 1.15.3 |
| Plotting-package versions | Not recorded in the retained environment metadata for this run |
| PyTorch and pytorch-tabnet | Used in earlier exploratory TabNet work; not required for the final candidate-model analysis |
| Random seed | Master seed 42; deterministic fold-specific seeds |
| Bootstrap repetitions | 1,000 |
| Primary architecture | Calibrated stacked ensemble |
| Base learners in locked stack | Penalized logistic regression, random forest, and HistGradientBoosting |
| Meta-learner | Logistic regression |
| Primary feature count | 25 |
| Exact locked threshold | 0.09438076500258265 |
| Threshold quantile | 0.90 |
| Model-selection AUROC tolerance | 0.01 |
| Model-selection Brier tie tolerance | 0.001 |
| Outer-fold assignment | Fixed and shared where required |
| Calibration-fold assignment | Fixed and shared where required |
| Validation threshold re-estimation | Disabled |
| Temporal-validation model refitting | Disabled |
| Geographic-validation model refitting | Disabled |
| Temporal-validation recalibration | Disabled |
| Geographic-validation recalibration | Disabled |

**Note:** The controlled institutional analysis archive retains the executable code, environment metadata, fold assignments, validation predictions, and quality-control files. Access is subject to institutional approval and applicable data-governance requirements. TabNet was evaluated only as an exploratory candidate and was not part of the locked primary stack.

**Supplementary Table S17. Distributions of calibrated model scores by age and educational-level category.**

**Panel A. Development cohort**

| Variable | Group | Encounters, n | Events, n | Event proportion, % | Mean score | Median score | P10 score | P90 score |
| --- | --- | --- | --- | --- | --- | --- | --- | --- |
| Age, years | <25 | 371 | 12 | 3.23 | 0.0314 | 0.0134 | 0.0067 | 0.0437 |
| Age, years | 25–34 | 697 | 19 | 2.73 | 0.0323 | 0.0110 | 0.0061 | 0.0524 |
| Age, years | 35–44 | 360 | 20 | 5.56 | 0.0599 | 0.0114 | 0.0062 | 0.1239 |
| Age, years | ≥45 | 157 | 22 | 14.01 | 0.1173 | 0.0393 | 0.0089 | 0.3124 |
| Education | Code 1 | 4 | 0 | 0.00 | 0.0652 | 0.0350 | 0.0082 | 0.1464 |
| Education | Code 2 | 16 | 1 | 6.25 | 0.0867 | 0.0202 | 0.0090 | 0.1642 |
| Education | Code 3 | 139 | 21 | 15.11 | 0.1357 | 0.0684 | 0.0101 | 0.3380 |
| Education | Code 4 | 484 | 21 | 4.34 | 0.0457 | 0.0104 | 0.0061 | 0.0767 |
| Education | Code 5 | 942 | 30 | 3.18 | 0.0335 | 0.0127 | 0.0064 | 0.0531 |

**Panel B. Temporal-validation cohort**

| **Variable** | **Group** | **Encounters, n** | **Events, n** | **Event proportion, %** | **Mean score** | **Median score** | **P10 score** | **P90 score** |
| --- | --- | --- | --- | --- | --- | --- | --- | --- |
| Age, years | <25 | 258 | 4 | 1.55 | 0.0240 | 0.0124 | 0.0079 | 0.0319 |
| Age, years | 25–34 | 354 | 7 | 1.98 | 0.0235 | 0.0113 | 0.0067 | 0.0378 |
| Age, years | 35–44 | 132 | 8 | 6.06 | 0.0634 | 0.0149 | 0.0067 | 0.0958 |
| Age, years | ≥45 | 59 | 10 | 16.95 | 0.1437 | 0.0262 | 0.0081 | 0.6005 |
| Education | Code 1 | 7 | 0 | 0.00 | 0.0162 | 0.0089 | 0.0077 | 0.0336 |
| Education | Code 3 | 33 | 7 | 21.21 | 0.1399 | 0.0482 | 0.0087 | 0.4522 |
| Education | Code 4 | 607 | 8 | 1.32 | 0.0280 | 0.0121 | 0.0072 | 0.0377 |
| Education | Code 5 | 156 | 14 | 8.97 | 0.0617 | 0.0136 | 0.0072 | 0.0895 |

**Panel C. Geographic external-validation cohort**

| Variable | Group | Encounters, n | Events, n | Event proportion, % | Mean score | Median score | P10 score | P90 score |
| --- | --- | --- | --- | --- | --- | --- | --- | --- |
| Age, years | <25 | 357 | 3 | 0.84 | 0.0217 | 0.0109 | 0.0071 | 0.0270 |
| Age, years | 25–34 | 732 | 17 | 2.32 | 0.0292 | 0.0114 | 0.0065 | 0.0381 |
| Age, years | 35–44 | 388 | 14 | 3.61 | 0.0517 | 0.0160 | 0.0068 | 0.0873 |
| Age, years | ≥45 | 145 | 18 | 12.41 | 0.1138 | 0.0271 | 0.0082 | 0.3717 |
| Education | Code 1 | 2 | 0 | 0.00 | 0.0294 | 0.0294 | 0.0215 | 0.0373 |
| Education | Code 2 | 8 | 1 | 12.50 | 0.0326 | 0.0197 | 0.0150 | 0.0656 |
| Education | Code 3 | 105 | 16 | 15.24 | 0.1340 | 0.0337 | 0.0114 | 0.6203 |
| Education | Code 4 | 558 | 16 | 2.87 | 0.0387 | 0.0115 | 0.0064 | 0.0468 |
| Education | Code 5 | 949 | 19 | 2.00 | 0.0312 | 0.0124 | 0.0067 | 0.0448 |

**Note:** Scores are from the selected stacked ensemble. Development scores were obtained by outer cross-fitting, including calibration. Validation scores were obtained from the model fitted on the full development cohort and applied without refitting or recalibration. Scores are reported on a 0–1 scale and should not be interpreted as exact individual probabilities. P10 and P90 denote the 10th and 90th percentiles. The educational-level codes used in this study were as follows: 1, illiterate; 2, primary school; 3, junior middle school; 4, senior middle school or technical secondary school; and 5, junior college or above. Codes are retained to support comparison with the source data. No temporal-validation encounters were recorded in educational-level category 2. Age and education are separate summaries of the same cohort and should not be added together. Each summary includes 1,585 encounters and 73 events in development, 803 encounters and 29 events in temporal validation, and 1,622 encounters and 52 events in geographic external validation. These descriptive results show score distributions across groups. They do not establish equal predictive performance or the absence of algorithmic bias. Estimates for small groups require caution.

**Supplementary Table S18. Paired bootstrap differences between calibrated candidate models in the development and validation cohorts.**

**Panel A. Development cohort: discrimination and prediction error**

| Model A | Model B | ΔAUROC (95% CI) | ΔAUPRC (95% CI) | ΔBrier score (95% CI) | ΔBSS (95% CI) |
| --- | --- | --- | --- | --- | --- |
| LR | HGB | −0.0150 (−0.0453, 0.0150) | 0.0224 (−0.0366, 0.0982) | −0.00141 (−0.00342, 0.00061) | 0.0322 (−0.0139, 0.0778) |
| LR | RF | −0.0533 (−0.0903, −0.0227) | −0.0619 (−0.1267, 0.0140) | 0.00182 (−0.00028, 0.00412) | −0.0414 (−0.0937, 0.0064) |
| LR | Stack | −0.0292 (−0.0498, −0.0128) | −0.0255 (−0.0758, 0.0347) | 0.00122 (−0.00070, 0.00306) | −0.0279 (−0.0696, 0.0160) |
| HGB | RF | −0.0383 (−0.0608, −0.0172) | −0.0843 (−0.1515, −0.0269) | 0.00323 (0.00148, 0.00512) | −0.0736 (−0.1166, −0.0336) |
| HGB | Stack | −0.0142 (−0.0342, 0.0062) | −0.0479 (−0.0963, −0.0047) | 0.00264 (0.00107, 0.00427) | −0.0600 (−0.0972, −0.0244) |
| RF | Stack | 0.0241 (0.0092, 0.0423) | 0.0364 (−0.0034, 0.0798) | −0.00059 (−0.00216, 0.00092) | 0.0135 (−0.0210, 0.0492) |

**Panel B. Development cohort: calibration**

| **Model A** | **Model B** | **ΔCITL (95% CI)** | **ΔJoint intercept (95% CI)** | **ΔCalibration slope (95% CI)** |
| --- | --- | --- | --- | --- |
| LR | HGB | 0.0950 (0.0088, 0.1801) | −0.1038 (−0.4126, 0.3078) | −0.0844 (−0.1987, 0.0599) |
| LR | RF | 0.0246 (−0.0618, 0.1131) | −0.1067 (−0.4604, 0.3093) | −0.0527 (−0.1836, 0.1007) |
| LR | Stack | −0.0139 (−0.0898, 0.0576) | −0.0100 (−0.3211, 0.3430) | 0.0079 (−0.0999, 0.1370) |
| HGB | RF | −0.0704 (−0.1382, −0.0028) | −0.0029 (−0.2122, 0.2317) | 0.0317 (−0.0577, 0.1247) |
| HGB | Stack | −0.1089 (−0.1768, −0.0383) | 0.0937 (−0.1317, 0.2921) | 0.0924 (0.0093, 0.1758) |
| RF | Stack | −0.0385 (−0.0975, 0.0205) | 0.0966 (−0.0928, 0.2682) | 0.0607 (−0.0220, 0.1424) |

**Panel C. Temporal-validation cohort: discrimination and prediction error**

| **Model A** | **Model B** | **ΔAUROC (95% CI)** | **ΔAUPRC (95% CI)** | **ΔBrier score (95% CI)** | **ΔBSS (95% CI)** |
| --- | --- | --- | --- | --- | --- |
| LR | HGB | 0.0100 (−0.0175, 0.0358) | −0.0046 (−0.0949, 0.0899) | −0.00133 (−0.00447, 0.00163) | 0.0381 (−0.0469, 0.1285) |
| LR | RF | −0.0162 (−0.0593, 0.0120) | −0.0961 (−0.1756, 0.0095) | 0.00172 (−0.00158, 0.00474) | −0.0494 (−0.1361, 0.0453) |
| LR | Stack | −0.0155 (−0.0475, 0.0041) | −0.0748 (−0.1561, −0.0081) | 0.00247 (−0.00036, 0.00531) | −0.0709 (−0.1525, 0.0104) |
| HGB | RF | −0.0262 (−0.0532, −0.0047) | −0.0915 (−0.1680, 0.0070) | 0.00305 (0.00075, 0.00549) | −0.0875 (−0.1578, −0.0215) |
| HGB | Stack | −0.0255 (−0.0468, −0.0067) | −0.0702 (−0.1280, −0.0199) | 0.00379 (0.00243, 0.00530) | −0.1090 (−0.1523, −0.0697) |
| RF | Stack | 0.0008 (−0.0094, 0.0120) | 0.0213 (−0.0525, 0.0649) | 0.00075 (−0.00072, 0.00233) | −0.0215 (−0.0669, 0.0208) |

**Panel D. Temporal-validation cohort: calibration**

| **Model A** | **Model B** | **ΔCITL (95% CI)** | **ΔJoint intercept (95% CI)** | **ΔCalibration slope (95% CI)** |
| --- | --- | --- | --- | --- |
| LR | HGB | −0.2421 (−0.4274, −0.0902) | −0.7738 (−1.5419, −0.0084) | −0.2550 (−0.6441, 0.0573) |
| LR | RF | −0.5567 (−0.7359, −0.4068) | −1.1112 (−1.8834, −0.4652) | −0.2099 (−0.5457, 0.0867) |
| LR | Stack | −0.4521 (−0.6134, −0.3168) | −1.0912 (−1.8190, −0.4466) | −0.2334 (−0.5096, 0.0454) |
| HGB | RF | −0.3146 (−0.4246, −0.1982) | −0.3374 (−0.9933, 0.2233) | 0.0451 (−0.2043, 0.3333) |
| HGB | Stack | −0.2100 (−0.2876, −0.1204) | −0.3174 (−0.8087, 0.0851) | 0.0216 (−0.1320, 0.2198) |
| RF | Stack | 0.1046 (0.0418, 0.1747) | 0.0200 (−0.4644, 0.5523) | −0.0236 (−0.2052, 0.1944) |

**Panel E. Geographic external-validation cohort: discrimination and prediction error**

| **Model A** | **Model B** | **ΔAUROC (95% CI)** | **ΔAUPRC (95% CI)** | **ΔBrier score (95% CI)** | **ΔBSS (95% CI)** |
| --- | --- | --- | --- | --- | --- |
| LR | HGB | −0.0077 (−0.0242, 0.0111) | −0.0771 (−0.1727, 0.0170) | 0.00071 (−0.00128, 0.00280) | −0.0227 (−0.0902, 0.0412) |
| LR | RF | −0.0240 (−0.0405, −0.0101) | −0.0381 (−0.1302, 0.0526) | 0.00026 (−0.00173, 0.00216) | −0.0085 (−0.0696, 0.0558) |
| LR | Stack | −0.0215 (−0.0342, −0.0103) | −0.0899 (−0.1686, −0.0034) | 0.00175 (0.00001, 0.00339) | −0.0564 (−0.1093, −0.0005) |
| HGB | RF | −0.0163 (−0.0357, −0.0012) | 0.0389 (−0.0347, 0.1154) | −0.00044 (−0.00243, 0.00151) | 0.0143 (−0.0486, 0.0783) |
| HGB | Stack | −0.0138 (−0.0317, −0.0007) | −0.0129 (−0.0483, 0.0232) | 0.00104 (−0.00023, 0.00229) | −0.0336 (−0.0736, 0.0073) |
| RF | Stack | 0.0025 (−0.0017, 0.0073) | −0.0518 (−0.1070, 0.0006) | 0.00149 (0.00018, 0.00277) | −0.0479 (−0.0893, −0.0058) |

**Panel F. Geographic external-validation cohort: calibration**

| **Model A** | **Model B** | **ΔCITL (95% CI)** | **ΔJoint intercept (95% CI)** | **ΔCalibration slope (95% CI)** |
| --- | --- | --- | --- | --- |
| LR | HGB | 0.1380 (0.0346, 0.2348) | −0.2905 (−0.9174, 0.3523) | −0.3252 (−0.6471, −0.0252) |
| LR | RF | −0.0481 (−0.1442, 0.0493) | 0.0941 (−0.3907, 0.6429) | 0.0049 (−0.2080, 0.2781) |
| LR | Stack | −0.0237 (−0.1164, 0.0695) | 0.0844 (−0.4035, 0.6546) | 0.0257 (−0.1704, 0.2870) |
| HGB | RF | −0.1861 (−0.2834, −0.0897) | 0.3846 (−0.1122, 0.8685) | 0.3301 (0.0930, 0.6251) |
| HGB | Stack | −0.1617 (−0.2288, −0.0903) | 0.3750 (0.0629, 0.7374) | 0.3509 (0.1859, 0.5951) |
| RF | Stack | 0.0244 (−0.0382, 0.0911) | −0.0097 (−0.2625, 0.2674) | 0.0208 (−0.0846, 0.1582) |

**Note:** Each entry reports the metric for Model A minus the metric for Model B, with its percentile 95% confidence interval. All comparisons use calibrated model scores. Development comparisons use outer cross-fitted predictions. Validation comparisons use predictions from models fitted on the development cohort and applied without refitting or recalibration.

Confidence intervals were calculated from 1,000 paired bootstrap samples. Encounters were resampled within outcome groups, and the same sampled encounters were used for both models in each comparison. All 1,000 requested samples produced valid estimates for every reported comparison. Predictions remained fixed during resampling. The intervals do not include uncertainty from refitting the full workflow. Development cross-fitted predictions also share training data, so these intervals are conditional and descriptive. The intervals are not adjusted for multiple comparisons.

Positive differences favor Model A for AUROC, AUPRC, and Brier skill score. Negative differences favor Model A for the Brier score. A positive or negative difference in a calibration parameter does not, by itself, establish better calibration. Calibration-in-the-large was estimated with the slope fixed at 1. The joint intercept and calibration slope were estimated together in a separate logistic recalibration model. Ideal values are 0 for calibration-in-the-large and the joint intercept, and 1 for the calibration slope. The individual model estimates should therefore be considered alongside these differences.

AUPRC was summarized using average precision. The primary model comparison was between HistGradientBoosting and the stacked ensemble. Comparisons involving penalized logistic regression and random forest provide additional evidence on model complexity and alternative candidates.

LR, penalized logistic regression; HGB, HistGradientBoosting; RF, random forest; Stack, stacked ensemble; AUROC, area under the receiver operating characteristic curve; AUPRC, area under the precision–recall curve; BSS, Brier skill score; CITL, calibration-in-the-large; CI, confidence interval.
